# Shufeng Jiedu Capsules for treating wind-heat syndrome respiratory diseases: a systematic review and meta-analysis

**DOI:** 10.3389/fphar.2025.1602563

**Published:** 2025-09-18

**Authors:** Jia-Min Liu, Lu Wang, Gui-Xiang Zhao, Hai-Long Zhang

**Affiliations:** ^1^ Department of Respiratory Diseases, The First Affiliated Hospital of Henan University of Chinese Medicine, Zhengzhou, China; ^2^ The First Clinical Medical College, Henan University of Chinese Medicine, Zhengzhou, China; ^3^ Collaborative Innovation Center for Chinese Medicine and Respiratory Diseases Co-Constructed by Henan Province and Education Ministry of P.R. China, Henan University of Chinese Medicine, Zhengzhou, China; ^4^ Henan Key Laboratory of Chinese Medicine for Respiratory Diseases, Henan University of Chinese Medicine, Zhengzhou, China

**Keywords:** Shufeng Jiedu Capsules, wind-heat syndrome, respiratory diseases, randomized controlled trials, meta-analysis

## Abstract

**Background:**

This study systematically evaluates the efficacy and safety of Shufeng Jiedu Capsules in treating respiratory diseases with wind-heat syndrome patterns, providing clinical guidance and a reference for developing new “syndrome-dominating disease management” medications.

**Methods:**

A systematic search of CNKI, Wanfang, VIP, SinoMed, PubMed, Cochrane Library, Embase, Web of Science, and trial registries identified randomized controlled trials (RCTs) assessing Shufeng Jiedu Capsules for wind-heat syndrome respiratory diseases (from inception to December 2024). Two researchers independently screened studies and extracted data using predefined criteria. Methodological quality was assessed using the Cochrane Risk of Bias tool. RevMan 5.3 and Stata 18 were used for data analysis, and evidence quality was graded using the GRADE system.

**Results:**

Twenty-five RCTs involving 2681 patients were included, with 1339 in the experimental group and 1342 in the control group. The GRADE assessment indicated predominantly low or very low evidence certainty, mainly due to methodological flaws such as unclear allocation concealment, lack of blinding, and absence of protocol registration, which increased the risk of bias. Meta-analysis showed that Shufeng Jiedu Capsule combined with conventional biomedical therapy outperformed biomedical therapy alone. It improved the effective rate of traditional Chinese medicine syndromes (risk ratio [RR] = 1.17, 95% CI 1.10 to 1.24, *P* < 0.00001) and shortened the resolution time of cough (mean difference [MD] = −0.97, 95% CI −1.09 to −0.85, *P* < 0.00001) and phlegm (MD = −0.48, 95% CI −0.96 to −0.17, *P* = 0.002). These outcomes were supported by high-quality GRADE evidence. Moderate-quality evidence supported improvements in imaging absorption rates (RR = 1.15, 95% CI 1.06 to 1.25, *P* = 0.0009) and shorter resolution time for pulmonary rales (MD = −1.48, 95% CI −2.86 to −0.10, *P* = 0.04). In contrast, apparent benefits in the clinical effective rate (RR = 1.16, 95% CI 1.12 to 1.19, *P* < 0.00001), fever resolution time (MD = −1.31, 95% CI −2.07 to −0.55, *P* = 0.0007), C-reactive protein levels (standardized MD [SMD] = −0.99, 95% CI −1.55 to −0.43, *P* = 0.0005), and procalcitonin levels (SMD = −2.06, 95% CI −3.62 to −0.49, *P* = 0.01) were based on low or very low certainty evidence. These results should be interpreted cautiously and require confirmation in rigorously designed trials. There was no significant difference in the incidence of adverse events between groups (RR = 0.88, 95% CI 0.34 to 2.31, *P* = 0.80). The adverse events were minor and controllable, and no serious adverse events were reported.

**Conclusion:**

Shufeng Jiedu Capsule combined with biomedicine may offer advantages in treating respiratory diseases with wind-heat syndrome. The adverse events were minor and controllable, and no serious adverse events were reported. However, the reliability of these findings is limited by low evidence certainty arising from methodological weaknesses in the included trials. High-quality, multicenter RCTs with rigorous methodology are essential to confirm these results.

**Systematic Review Registration:**

https://www.crd.york.ac.uk/PROSPERO/view/CRD420251017879, identifier CRD420251017879.

## 1 Introduction

Acute respiratory diseases remain a global health challenge, with wind-heat syndrome being a primary pattern in traditional Chinese medicine (TCM) for conditions such as influenza and acute exacerbations of chronic obstructive pulmonary disease (AECOPD) (Zhang J. P., 2022; Pan et al., 2020). Biomedical therapies generally target pathogen eradication but often overlook the dynamic progression driven by TCM syndromes, creating a key opportunity for TCM interventions. Syndrome differentiation is a prerequisite for TCM diagnosis and treatment. The integrated disease–syndrome diagnosis and treatment model provides a vital framework for TCM and integrated Chinese–biomedical research. Clinical studies often combine Western disease diagnoses with TCM syndrome classifications. This model includes two forms: “disease-centered syndrome management” and “syndrome-dominating disease management” (Li, 2011). Compared with the disease-centered approach, the syndrome-dominating model better reflects TCM’s core diagnostic philosophy, which emphasizes syndrome differentiation. It also aligns more closely with the dynamic disease progression patterns observed in real-world TCM practice. As a supplement to the disease–syndrome integration model, the syndrome-dominating approach is gaining increasing recognition (Zhao J. L. et al., 2018). However, no systematic review or meta-analysis of commercial Chinese polyherbal preparations (CCPPs) for wind-heat syndrome has yet been conducted under this framework.

The biomedical correlates of wind-heat syndrome in TCM include acute fever, inflammation, sore throat, and cough (Liu et al., 2022). These clinical features closely parallel those observed in acute respiratory tract infections (ARTIs), such as acute upper respiratory tract infection (AURTI) and community-acquired pneumonia (CAP). Therefore, methodologically, it is essential to evaluate the efficacy of TCM interventions for wind-heat syndrome within the context of biomedically defined ARTIs. This approach establishes a scientifically testable paradigm, allowing traditional medical concepts to be objectively evaluated using clinical outcomes such as the duration until fever resolution, reduction in inflammatory biomarkers (e.g., C-reactive protein, procalcitonin), and symptom severity improvement. Accordingly, this systematic review uses ARTIs as a clinically relevant model to investigate the efficacy of the commercial Chinese polyherbal preparation (CCPP) Shufeng Jiedu Capsules for treating wind-heat syndrome.

Oral CCPPs are widely used in clinical practice because they are portable and easy to administer. Shufeng Jiedu Capsule (SFJDC), a widely used CCPP, is derived from the century-old ancestral formula “Qudu San” (Ou and Ning, 2017), which was donated to Chu Xian County by a folk TCM practitioner from Xiangxi, Hunan Province. The formula is designed to synergistically clear heat-toxin, strengthen healthy qi to expel pathogens, promote light-dispersing diffusion, and detoxify to relieve pharyngitis (Zhu et al., 2022). In recent years, Shufeng Jiedu Capsule has been extensively used to treat respiratory diseases such as influenza, ARTIs, AECOPD, tracheobronchitis, and CAP (Ou and Ning, 2017; Luo et al., 2023; Li J. B. et al., 2023; Yin et al., 2022). Multiple clinical studies have confirmed its therapeutic efficacy in respiratory infections with wind-heat syndrome (Xu et al., 2015). Comprehensive clinical evaluation of CCPPs is a key focus in current TCM assessment research (Liao et al., 2022). However, current evidence lacks a syndrome-oriented evaluation of Shufeng Jiedu Capsules for respiratory diseases, limiting its potential optimization in clinical practice. This study addresses this gap by conducting a systematic review and meta-analysis under the syndrome-dominating framework to evaluate the clinical efficacy of Shufeng Jiedu Capsules for wind-heat syndrome respiratory diseases. The findings aim to quantify syndrome-specific efficacy, establish a new paradigm for TCM clinical evaluation, and provide evidence to support syndrome-driven drug development.

## 2 Methods

This review was reported according to the Preferred Reporting Items for Systematic Reviews and Meta-Analyses (PRISMA) reporting guidelines (Page et al., 2021) The study protocol was registered in PROSPERO (Registration number: CRD420251017879).

### 2.1 Data sources and search strategy

We searched the following databases: CNKI, Wanfang, VIP, SinoMed, PubMed, Cochrane Library, Embase, and Web of Science. The search period covered database inception to December 2024. Only studies published in Chinese or English were considered. The search included journals, graduate theses, newspapers, and conference proceedings. The literature term was conducted using a combination of subject words and free-text words, including “Shufeng Jiedu Capsules”, “Shufeng Jiedu”, “Shu Feng Jie Du Capsules”, “ShufengJiedu”. Detailed search strategies are provided in the Supplementary Table S1.

### 2.2 Inclusion criteria

The inclusion criteria were defined according to the PICOS framework. (1) Population: Adult patients with a confirmed diagnosis of wind-heat syndrome or wind-heat invading the lung syndrome, which biomedically manifests as respiratory diseases, including Acute Upper Respiratory Tract Infection (AURTI), Acute Exacerbation of Chronic Obstructive Pulmonary Disease (AECOPD), Acute Exacerbation of Chronic Bronchitis (AECB), Acute Tracheobronchitis (AT), Community-Acquired Pneumonia (CAP), or other related respiratory conditions. Studies were selected because these biomedical conditions represent the most prevalent and clinically relevant manifestations of wind-heat syndrome, providing a measurable framework for assessing the efficacy of the botanical drug. (2) Intervention: Administration of Shufeng Jiedu Capsules as the experimental treatment. (3) Comparison: The control group received standard biomedicine therapy, including antitussive, expectorant, anti-infective, and other symptomatic supportive treatments. The experimental group received Shufeng Jiedu Capsules, a commercial Chinese polyherbal preparation, in addition to the same biomedicine therapy. (4) Outcomes: Studies were required to report at least one of the following: Clinical efficacy rate, TCM syndrome efficacy, resolution time of clinical symptoms/signs (fever, cough, sputum, pulmonary rales), inflammatory biomarkers (C-reactive protein CRP, procalcitonin PCT), imaging absorption rate, or adverse events. (5) Study design: RCTs.

Shufeng Jiedu Capsule is an over-the-counter CCPP approved by the National Medical Products Administration (NMPA) of China (NMPA, 2021). Its standardized formulation is well-defined in pharmacopeial standards and consists of eight botanical drugs: *Bupleurum chinense DC.* [*Apiaceae*; *Bupleuri Radix*], *Forsythia suspensa (Thunb.) Vahl* [*Oleaceae*; *Forsythiae Fructus*], *Glycyrrhiza uralensis Fisch. ex DC.* [*Fabaceae*; *Glycyrrhizae Radix Et Rhizoma*], *Isatis indigotica subsp. tinctoria* [*Brassicaceae*; *Isatidis Radix*], *Patrinia scabiosifolia Link* [*Caprifoliaceae*; *Patriniae Herba*], *Phragmites australis (Cav.) Trin. ex Steud.* [*Poaceae*; *Phragmitis Rhizoma*], *Reynoutria japonica Houtt.* [*Polygonaceae*; *Polygoni Cuspidati Rhizoma et Radix*], and *Verbena officinalis L.* [*Verbenaceae*; *Verbenae Herba*]. The complete, taxonomically validated composition is provided in Supplementary Table S2. Detailed reporting of its composition in the primary studies included in this systematic review is summarized in Supplementary Table S3. All botanical drugs were validated using the Medicinal Plant Names Services (MPNS) and Plants of the World Online (POWO). The ConPhyMP checklist was completed to ensure comprehensive reporting (Supplementary Appendix A3).

### 2.3 Exclusion criteria

The exclusion criteria were as follows: (1) Studies lacking accessible full-texts or sufficient extractable data; (2) Duplicate publications; (3) Studies that did not clearly specify the TCM syndrome as Wind-Heat syndrome; (4) Studies in which interventions did not involve capsule formulations.

### 2.4 Study selection and data extraction

Firstly, the retrieved results were imported into EndNote X8 software, duplicates were removed, and preliminary screening was performed by reviewing titles and abstracts. Full-texts of the screened studies were retrieved and assessed for eligibility, with studies not meeting the inclusion criteria excluded. Data from the final included studies were extracted using Excel 2020, including first author, publication year, sample size, intervention details, diagnostic criteria for traditional Chinese and biomedicine, randomization method, blinding, outcome measures, and relevant diagnostic criteria. All screening and data extraction were performed independently by at least two researchers (LW and GXZ). Discrepancies were resolved through discussion or consultation with a third author (JML).

### 2.5 Risk of bias assessment

The risk of bias in the included studies was assessed using the Cochrane Handbook “Risk of Bias” tool (Sterne et al., 2019), with two researchers independently evaluating seven key domains: (1) the method of generating random sequences. (2) whether allocation concealment was implemented. (3) whether blinding was applied to participants and personnel. (4) whether outcome assessors were blinded. (5) whether outcome reporting was complete. (6) whether selective reporting was present. (7) Whether other sources of bias potentially affecting the authenticity of the findings existed. Ultimately, the risk of bias for each study was classified as “low”, “high”, or “unclear”. Disagreements were resolved through discussion or consultation with a third reviewer.

### 2.6 Statistical analysis

Statistical analyses were performed using Review Manager version 5.3 and STATA 18. For continuous variables, mean differences (MDs) with 95% confidence intervals (CIs) were used as effect measures. For dichotomous variables, risk ratios (RRs) with 95% CIs were applied. When measurement units were inconsistent, values were converted to standardized mean differences (SMDs) with 95% CIs. Heterogeneity between studies was assessed using the I^2^ statistic. When heterogeneity was low (*I* ^*2*^ < 50%, *P* > 0.05), a fixed-effects model was applied. When heterogeneity was high (*I* ^*2*^ > 50%, *P* < 0.05), a random-effects model was used. Subgroup and sensitivity analyses were conducted to explore potential sources of heterogeneity. If heterogeneity remained excessive or its sources could not be identified, only descriptive analysis was performed. Publication bias was assessed using funnel plots and Egger’s regression test.

### 2.7 Quality of evidence

The Grading of Recommendations Assessment, Development, and Evaluation (GRADE) system (Liu, 2022) was used to assess evidence quality across five domains: risk of bias, inconsistency, indirectness, imprecision, and publication bias. Evidence was classified into four grades: high, moderate, low, or very low.

## 3 Results

### 3.1 Search results

**FIGURE 1:**
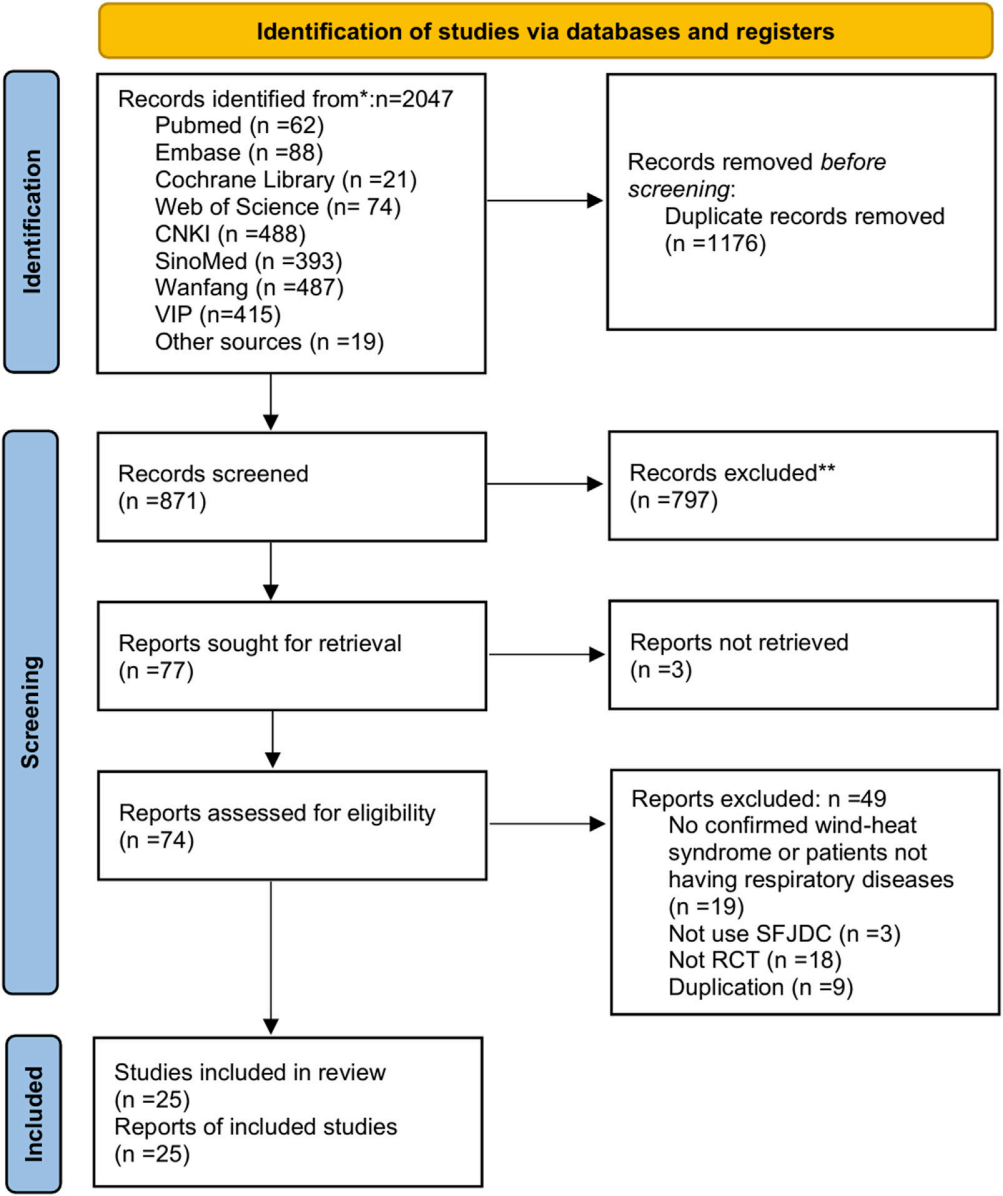
PRISMA flow chart of study selection.

The initial search identified 2047 articles. After removing 1176 duplicates, 871 articles remained. Screening of titles and abstracts led to the exclusion of 797 articles, leaving 77 for full-text review. Three reports could not be retrieved and were excluded. Of the remaining 74 articles, 49 were excluded for the following reasons: Population: 19 studies lacked confirmed wind-heat syndrome diagnosis or included non-respiratory disease patients. Intervention: 3 studies did not use Shufeng Jiedu Capsules as the primary intervention. Study design: 18 were non-randomized controlled trials (RCTs). Duplicates: 9 were duplicate publications not detected in the initial screening. The screening process adhered strictly to predefined PICOS criteria and established inclusion/exclusion protocols. A total of 25 studies (Li, 2023; Li, 2022; Li and Zhang L. S., 2022; Zhang J. P., 2022; Chen and Zhang, 2021; Tang et al., 2021; Xia et al., 2021; Fei et al., 2020; Pan et al., 2020; Zhang and Zhang, 2020; Zhao and Wang, 2020; Wang and Zhao, 2020; Shen, 2021; Yang et al., 2019; He, 2019; Zhou et al., 2019; Wu et al., 2019; Chang, 2019; Li and Li, 2019; Zhu et al., 2018; Zhao C. et al., 2018; Xie, 2017; Wei et al., 2016; Han et al., 2016; Zhang et al., 2014) met the criteria and were included in the analysis. All included studies were published in Chinese. The screening process is illustrated in Figure 1.

### 3.2 Description of the included studies

The 25 included studies involved 2681 patients, with 1339 in the experimental group and 1342 in the control group. All studies addressed wind-heat syndrome or wind-heat invading lung syndrome. The disease distribution was as follows: six studies (Li, 2022; Zhang and Zhang, 2020; Zhao and Wang, 2020; Wang and Zhao, 2020; Zhao J. L. et al., 2018; Zhang et al., 2014) on acute upper respiratory tract infections, seven on CAP (Li and Zhang, 2022; Tang et al., 2021; Xia et al., 2021; Pan et al., 2020; Zhou et al., 2019; Wu et al., 2019; Wei et al., 2016), 9 on AECOPD (Li, 2023; Chen and Zhang, 2021; Fei et al., 2020; Shen, 2021; Yang et al., 2019; He, 2019; Chang, 2019; Li and Li, 2019; Zhu et al., 2018), two on acute exacerbations of chronic bronchitis (AECB) (Xie, 2017; Han et al., 2016), and 1 on acute tracheobronchitis (Zhang J. P., 2022). In all trials, the experimental group received Shufeng Jiedu Capsules combined with biomedicine. The control group received biomedicine alone. Biomedical interventions mainly consisted of symptomatic and supportive treatments, including anti-infective therapy, cough relief, and phlegm reduction. The basic characteristics of the included studies are presented in Table 1.

**TABLE 1:** Basic characteristics of included articles.

Study	Sample size	Age (years old)	Gender (male/female)		Intervention		Course of treatment (days)	Diseases	Outcomes
	E/C	E/C	E	C	E	C			
Li (2023)	30/30	69.60 ± 6.48/69.66 ± 6.27	21/9	19/11	SFJDC + WM	WM	7	(1)	A, B
Li (2022)	50/50	30.16 ± 5.62/29.63 ± 5.28	26/24	23/27	SFJDC + WM	WM	3	(2)	A, G
Li and Zhang (2022)	35/35	68.57 ± 5.36/67.35 ± 5.81	16/19	17/18	SFJDC + WM	WM	7	(3)	A, C, D, E, F, G, H, J
Zhang J. P. (2022)	60/60	43.65 ± 9.44/48.59 ± 11.21	31/29	26/34	SFJDC + WM	WM	5	(5)	A, G, J
Chen and Zhang (2021)	30/30	65.79 ± 8.40/63.21 ± 7.15	25/5	20/10	SFJDC + WM	WM	10	(1)	J
Tang et al. (2021)	60/60	52.19 ± 5.25/53.23 ± 5.11	37/23	34/26	SFJDC + WM	WM	7	(3)	A, C, D, E, F, G, J
Xia et al. (2021)	60/60	70.93 ± 5.54/70.93 ± 5.54	31/29	33/27	SFJDC + WM	WM	8	(6)	A, G, H
Shen (2021)	50/50	74.46 ± 9.66/74.46 ± 9.66	37/13	39/11	SFJDC + WM	WM	6	(1)	A, B, J, H
Fei et al. (2020)	56/56	55.16 ± 7.35/55.24 ± 7.43	29/27	32/24	SFJDC + WM	WM	14	(4)	A, E, F, G
Pan et al. (2020)	60/60	39.81 ± 10.95/38.87 ± 10.28	36/24	32/28	SFJDC + WM	WM	10	(3)	I, J
Zhang and Zhang (2020)	118/119	31.94 ± 6.50/35.94 ± 3.50	67/51	65/54	SFJDC + WM	WM	3	(2)	A
Zhao and Wang (2020)	78/78	37.30 ± 6.50/36.80 ± 6.20	41/37	40/38	SFJDC + WM	WM	5	(2)	A, C, J
Wang and Xie (2020)	49/49	44.38 ± 11.27/46.14 ± 10.21	28/21	27/22	SFJDC + WM	WM	5	(7)	A, C, D, J
Yang et al. (2019)	50/50	61.30 ± 4.70/63.15 ± 3.71	27/23	24/26	SFJDC + WM	WM	7	(1)	A, B, G, H, J
He (2019)	30/30	63.21 ± 7.15/65.79 ± 8.40	25/5	20/10	SFJDC + WM	WM	10	(1)	A, B, J
Zhou et al. (2019)	40/40	50.30 ± 6.40/48.56 ± 5.40	24/16	22/18	SFJDC + WM	WM	7	(3)	A, C, D, E, F, G, J
Wu et al. (2019)	86/86	38.63 ± 7.09/36.18 ± 8.10	0/86	0/86	SFJDC + WM	WM	7	(3)	A, C, F, G, J
Chang (2019)	32/32	74.92 ± 6.57/75.13 ± 6.62	20/12	21/11	SFJDC + WM	WM	7	(1)	A
Li and Li (2019)	40/40	72.56 ± 5.46/72.26 ± 5.68	26/14	28/12	SFJDC + WM	WM	7	(1)	A
Zhu et al. (2018)	30/30	52–89/60–89	19/11	21/9	SFJDC + WM	WM	6	(1)	A, B, J
Zhao J. L. et al. (2018)	61/62	45.69 ± 11.30/45.21 ± 10.36	30/31	33/29	SFJDC + WM	WM	5	(2)	A
Xie (2017)	45/45	57.5 ± 15.7/57.1 ± 16.8	25/20	27/18	SFJDC + WM	WM	7–14	(4)	A, B, D, E, F, I, J
Wei et al. (2016)	60/60	57.64 ± 7.35/56.84 ± 8.13	32/28	31/29	SFJDC + WM	WM	7	(3)	A, B, C, D, G, H, I, J
Han et al. (2016)	34/34	56.10 ± 6.70/54.50 ± 7.20	25/9	27/7	SFJDC + WM	WM	10	(4)	A, J
Zhang et al. (2014)	110/110	40.02 ± 3.18/39.81 ± 3.42	60/50	65/55	SFJDC + WM	WM	NR	(2)	A

E, Experimental group; C, Control group; NR, Not Report; SFJDC, Shufeng Jiedu Capsules; WM, Western medical treatment; (1), AECOPD; (2), AURTI; (3), CAP; (4), AECB; (5), AT; (6), AECOPD-CAP; (7), Influenza Complicated with Acute Tonsillitis; A, Clinical effective rate; B, effective rate of TCM, syndromes; C, resolution time of fever; D, resolution time of cough; E, resolution time of phlegm; F, resolution time of pulmonary rales; G, CRP; H, PCT; I, imaging absorption rate; J, adverse events.

### 3.3 Risk of bias assessment of included studies

**FIGURE 2:**
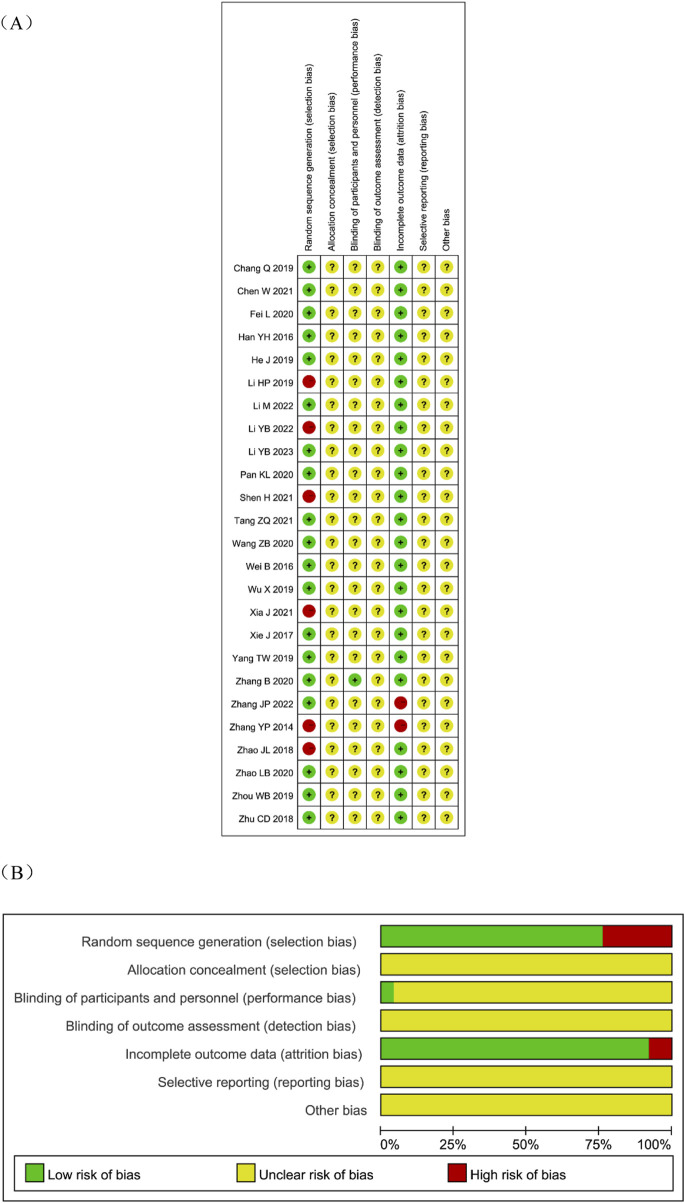
Risk of bias assessment for eligible studies. **(A)** Risk of bias summary; **(B)** Risk of bias graph.

Nineteen studies reported randomization. Of these, 17 used random number tables (Li, 2023; Li, 2022; Zhang J. P., 2022; Chen and Zhang, 2021; Tang et al., 2021; Fei et al., 2020; Pan et al., 2020; Zhang and Zhang, 2020; Zhao and Wang, 2020; Wang and Zhao, 2020; Yang et al., 2019; He, 2019; Zhou et al., 2019; Chang, 2019; Xie, 2017; Wei et al., 2016; Han et al., 2016), 1 used coin tossing (Zhu et al., 2018), and 1 used random drawing (Wu et al., 2019). Six studies (Li and Zhang, 2022; Xia et al., 2021; Shen, 2021; Li and Li, 2019; Zhao J. L. et al., 2018; Zhang et al., 2014) mentioned randomization without specifying the method. None of the studies reported allocation concealment, and this was therefore rated as unclear. Only one study (Zhang and Zhang, 2020) described blinding, which was assessed as low risk; the others provided no relevant details and were rated as unclear. No study described blinding of outcome assessment, which was also rated as unclear. Two studies (Zhang J. P., 2022; Zhang et al., 2014) reported loss to follow-up and were assessed as high risk for attrition bias. None of the studies were registered in a protocol registry, so selective reporting risk was rated as unclear. Other potential sources of bias could not be determined and were rated as unclear. The detailed risk of bias assessment is shown in Figure 2. A considerable proportion of the included trials were judged to have an “unclear” risk of bias in critical domains, such as random sequence generation, allocation concealment, and blinding. The pervasive “unclear” reporting inherently limits the strength of evidence, potentially leading to an overestimation of treatment effects due to performance and selection biases.

### 3.4 Primary outcomes

#### 3.4.1 Clinical effective rate

**FIGURE 3:**
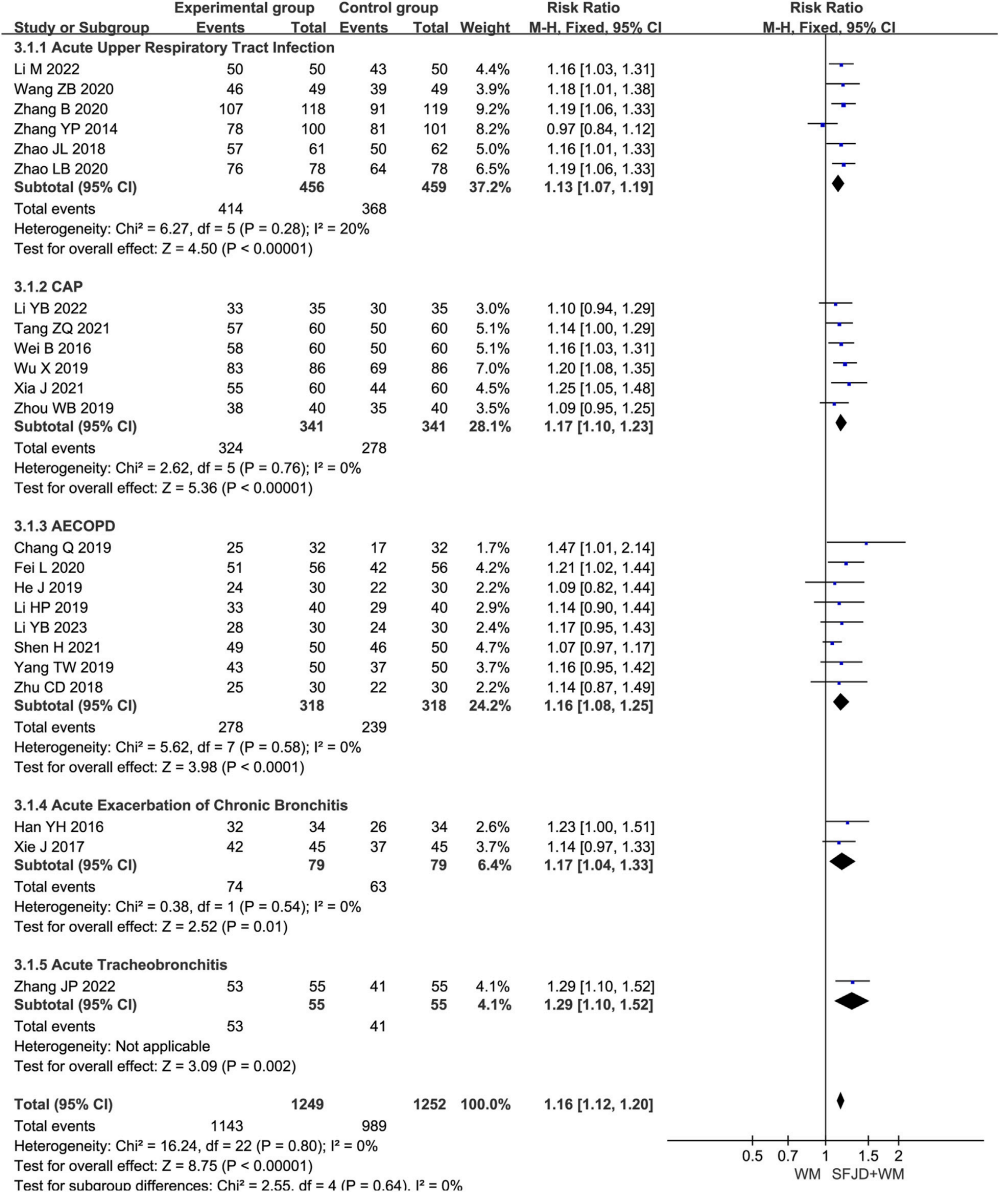
Forest plot of Clinical effective rate between experimental group and control group.

A total of 23 articles (Li, 2023; Li, 2022; Li and Zhang, 2022; Zhang J. P., 2022; Tang et al., 2021; Xia et al., 2021; Fei et al., 2020; Zhang and Zhang, 2020; Zhao and Wang, 2020; Wang and Zhao, 2020; Shen, 2021; Yang et al., 2019; He, 2019; Zhou et al., 2019; Wu et al., 2019; Chang, 2019; Li and Li, 2019; Zhu et al., 2018; Zhao C. et al., 2018; Xie, 2017; Wei et al., 2016; Han et al., 2016; Zhang et al., 2014) reported the overall clinical efficacy rate, involving 2501 patients. The heterogeneity test result was *P* = 0.73 and *I* ^*2*^ = 0%. As there was no heterogeneity, a fixed-effects model was used for the meta-analysis. The findings suggested that, compared with conventional biomedical therapy alone, Shufeng Jiedu Capsule combined with biomedical therapy may be associated with improved clinical effective rates in patients with respiratory diseases presenting with wind-heat syndrome (RR = 1.16, 95% CI 1.12 to 1.19, *P* < 0.00001). Given the low certainty of evidence, these findings require verification in high-quality studies. Subgroup analysis by respiratory disease type yielded results consistent with the overall findings and showed low heterogeneity (Figure 3). Sensitivity analysis confirmed the robustness and reliability of the conclusions (Supplementary Figure S11A).

#### 3.4.2 Effective rate of TCM syndromes

**FIGURE 4:**
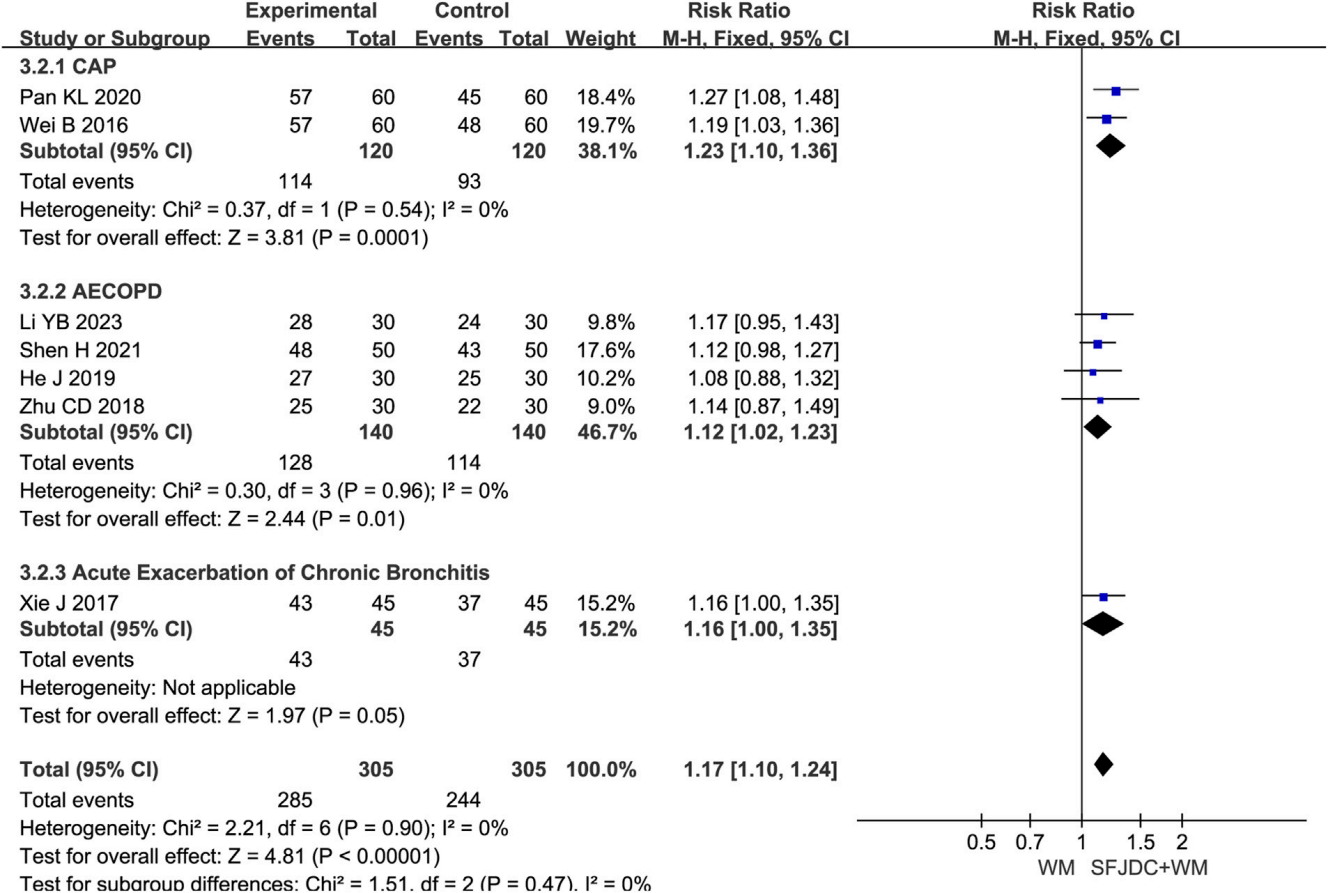
Forest plot of Effective rate of TCM syndromes between experimental group and control group.

Seven articles (Li, 2023; Pan et al., 2020; Shen, 2021; He, 2019; Zhu et al., 2018; Xie, 2017; Wei et al., 2016) involving 610 patients reported the effective rate of TCM syndromes using unified reference standards (National Medical Products Administration, 2002). The heterogeneity test result was *P* = 0.90 and *I* ^*2*^ = 0%. A fixed-effects model was therefore applied. Compared with conventional biomedical therapy alone, Shufeng Jiedu Capsule combined with biomedical therapy appeared to improve the effective rate of TCM syndromes in patients with respiratory diseases and wind-heat syndrome (RR = 1.17, 95% CI 1.10 to 1.24, *P* < 0.00001). Sensitivity analysis confirmed the robustness and reliability of the conclusions (Supplementary Figure S11B). Subgroup analysis by respiratory disease type produced results consistent with the overall analysis and showed no heterogeneity (Figure 4).

#### 3.4.3 Resolution time of fever

**FIGURE 5:**
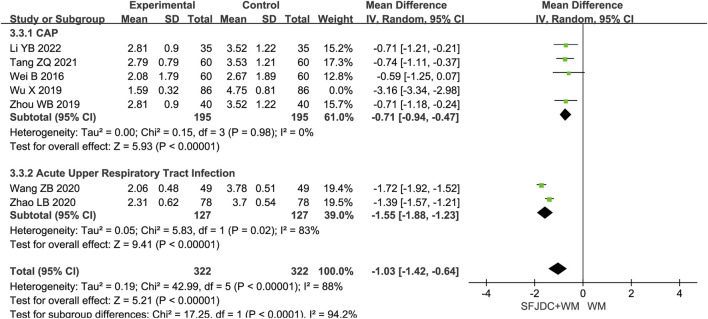
Forest plot of Resolution time of fever between experimental group and control group.

Seven articles (Li and Zhang, 2022; Tang et al., 2021; Zhao and Wang, 2020; Wang and Zhao, 2020; Zhou et al., 2019; Wu et al., 2019; Wei et al., 2016), involving 816 patients, reported on the resolution time of fever. The heterogeneity test result was *P* < 0.00001, *I* ^*2*^ = 98%. Due to the high heterogeneity, a random-effects model was applied. The results suggested that, compared with conventional biomedical therapy alone, Shufeng Jiedu Capsule combined with biomedical therapy may reduce fever resolution time in patients with respiratory diseases and wind-heat syndrome (MD = −1.31, 95% CI -2.07 to −0.55, *P* = 0.0007). Given the low certainty of evidence, these results require confirmation through high-quality studies. Subgroup analyses by age and publication year were consistent with the overall findings, although high heterogeneity persisted (Supplementary Figure S1). Subgroup analysis by respiratory disease type showed that the acute upper respiratory tract infection group had results consistent with the overall findings, whereas the CAP group showed no significant reduction in fever resolution time. Sensitivity analysis, conducted by sequentially excluding individual studies, revealed that removing Wu Xia (Wu et al., 2019) in the CAP group reduced *I* ^*2*^ from 98% to 0% and markedly altered the results. The primary difference in Wu Xia’s study (Wu et al., 2019) was that all patients were female, which may explain the high heterogeneity and unstable results (Figure 5 shows the forest plot after removal; Supplementary Figure S2 shows it before removal).

#### 3.4.4 Resolution time of cough

**FIGURE 6:**
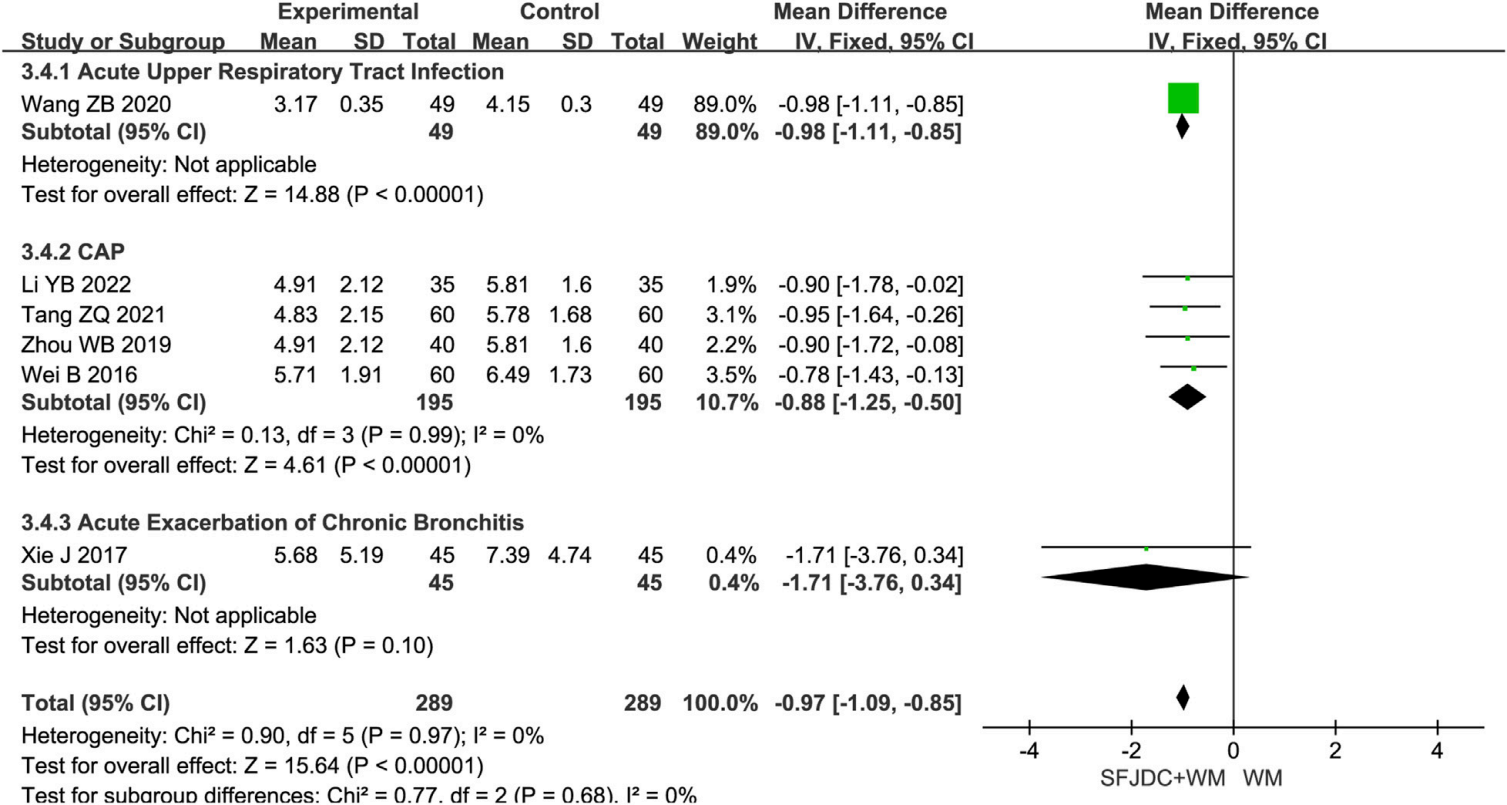
Forest plot of Resolution time of cough between experimental group and control group.

Six articles (Li and Zhang, 2022; Tang et al., 2021; Wang and Zhao, 2020; Wei et al., 2016), involving 578 patients, reported the resolution time of cough. The heterogeneity test result was *P* = 0.97, *I* ^*2*^ = 0%. A fixed-effects model was applied. Compared with conventional biomedical therapy alone, Shufeng Jiedu Capsule combined with biomedical therapy significantly reduced cough resolution time in patients with respiratory diseases and wind-heat syndrome (MD = −0.97, 95% CI -1.09 to −0.85, *P* < 0.00001). Sensitivity analysis confirmed the robustness of the results (Supplementary Figure S11C). Subgroup analysis by disease type showed inconsistent results for the AECB group; however, this subgroup was based on a single study, and confirmation in large multicenter trials is needed (Figure 6).

#### 3.4.5 Resolution time of phlegm

**FIGURE 7:**
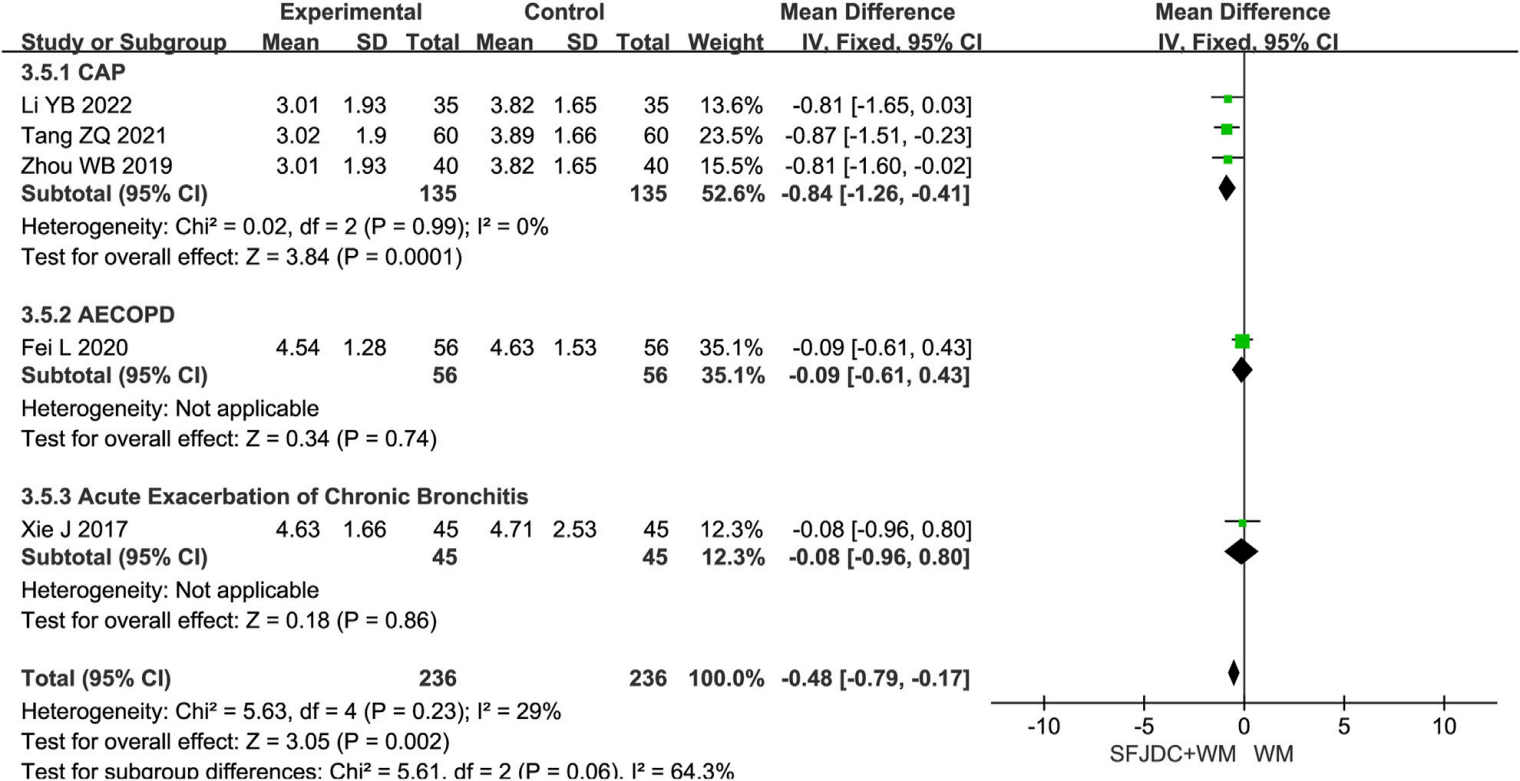
Forest plot of Resolution time of phlegm between experimental group and control group.

Five articles (Li and Zhang, 2022; Tang et al., 2021; Fei et al., 2020; Zhou et al., 2019; Xie, 2017), involving 472 patients, reported the resolution time of phlegm. The heterogeneity test result was *P* = 0.23 and *I* ^*2*^ = 29%. As heterogeneity was low, a fixed-effects model was used. The findings indicated that, compared with conventional biomedical therapy alone, Shufeng Jiedu Capsule combined with biomedical therapy shortened phlegm resolution time in patients with respiratory diseases and wind-heat syndrome (MD = −0.48, 95% CI -0.96 to −0.17, *P* = 0.002). Sensitivity analysis confirmed result stability (Supplementary Figure S11D). Subgroup analysis by disease type showed consistent results with no heterogeneity for the CAP subgroup. However, the AECOPD and AECB subgroups had results inconsistent with the overall findings. Both subgroups were based on single studies, and further large-scale multicenter research is needed (Figure 7).

#### 3.4.6 Resolution time of pulmonary rales

**FIGURE 8:**
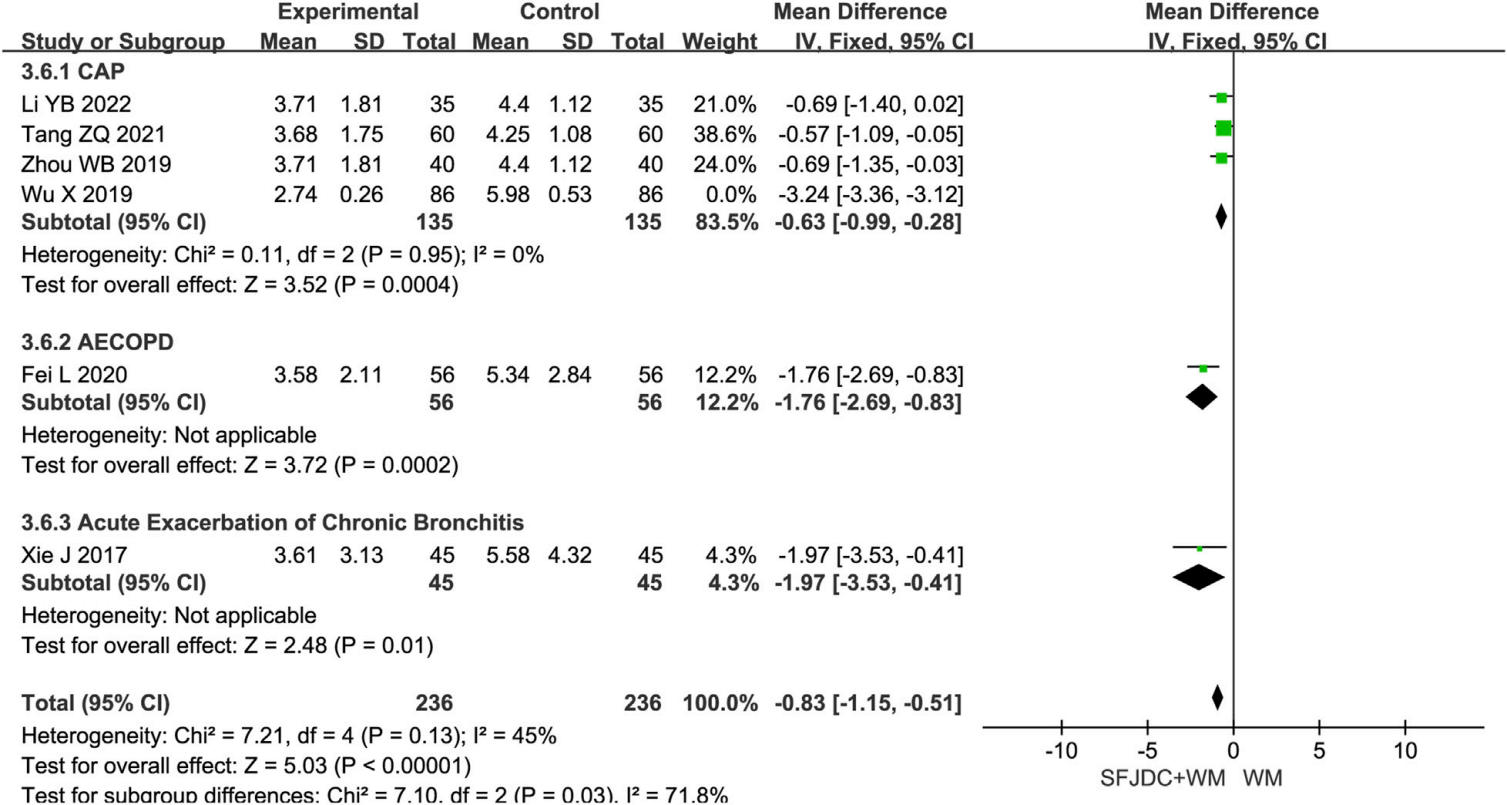
Forest plot of Resolution time of pulmonary rales between experimental group and control group.

Six articles (Li and Zhang, 2022; Tang et al., 2021; Fei et al., 2020; Zhou et al., 2019; Wu et al., 2019; Xie, 2017), involving 472 patients, reported the resolution time of pulmonary rales. The heterogeneity test showed *P* < 0.00001 and *I* ^*2*^ = 97%, indicating substantial heterogeneity. Consequently, a random-effects model was applied in the meta-analysis. Compared with conventional biomedical therapy alone, Shufeng Jiedu Capsules combined with biomedical therapy may shorten the duration of pulmonary rales in patients with respiratory diseases presenting with wind-heat syndrome (MD = −1.48, 95% CI -2.86 to −0.10, *P* = 0.04). Subgroup analyses stratified by publication year, age, and treatment duration yielded results consistent with the overall pooled estimates, although high heterogeneity persisted across all subgroups (Supplementary Figure S3). Further subgroup analysis based on respiratory disease types revealed that, in the CAP subgroup, the combination of Shufeng Jiedu Capsules with conventional biomedical therapy did not significantly reduce the resolution time of pulmonary rales compared with conventional biomedical therapy group. Due to the high heterogeneity, sensitivity analysis was conducted by sequentially excluding individual studies. After excluding Wu Xia’s study (Wu et al., 2019), the *I* ^*2*^ value in the CAP group decreased markedly from 98% to 0%, indicating that heterogeneity was significantly reduced and the pooled results were altered. The main distinction in Wu Xia’s study (Wu et al., 2019) ’s study compared with the others was that all included patients included were female, which may account for the large heterogeneity and unstable results. Figure 8 presents the forest plot after excluding this study, and Supplementary Figure S4 shows the forest plot before exclusion.

### 3.5 Secondary outcomes

#### 3.5.1 Inflammatory factors: C-reactive protein

Ten articles (Li, 2022; Li and Zhang, 2022; Zhang L. S., 2022; Tang et al., 2021; Xia et al., 2021; Fei et al., 2020; Yang et al., 2019; Zhou et al., 2019; Wu et al., 2019; Wei et al., 2016), involving 1104 patients, reported data on CRP. The heterogeneity test yielded *P* < 0.00001 and *I* ^*2*^ = 95%, indicating high heterogeneity. A random-effects model was applied for meta-analysis. The pooled results suggested that compared with conventional biomedical therapy alone, Shufeng Jiedu Capsule combined with biomedical therapy may reduce CRP levels in patients with respiratory diseases presenting with wind-heat syndrome (SMD = −0.99, 95% CI -1.55 to −0.43, *P* = 0.0005). However, the certainty of this evidence was very low, and further well-designed trials are needed to confirm these findings. Subgroup analyses stratified by publication year, age, and treatment duration yielded results consistent with the overall pooled estimates, although high heterogeneity persisted across all subgroups (Supplementary Figure S5). When stratified by disease type, the results for the AECOPD subgroup differed from the overall meta-analysis results but were consistent with the other disease-specific subgroups. Nonetheless, the AECOPD group results require validation through large-scale multicenter studies (Supplementary Figure S6). Sensitivity analysis confirmed the robustness and reliability of the conclusions (Supplementary Figure S11E).

#### 3.5.2 Inflammatory factors: procalcitonin

Five articles (Li and Zhang, 2022; Shen, 2021; Yang et al., 2019; Xia et al., 2021; Wei et al., 2016), involving 510 patients, reported on PCT levels. The heterogeneity test indicated *P* < 0.00001 and *I* ^*2*^ = 98%, reflecting high heterogeneity. A random-effects model was applied for the meta-analysis. Compared with conventional biomedical therapy alone, Shufeng Jiedu Capsules combined with biomedical therapy may reduce PCT levels in patients with respiratory diseases presenting with wind-heat syndrome (SMD = −2.06, 95% CI -3.62 to −0.49, *P* = 0.01). Due to the very low certainty of evidence, these results require confirmation in rigorously designed clinical trials. Subgroup analyses stratified by publication year showed outcomes consistent with the overall pooled results, although high heterogeneity persisted across the subgroups (Supplementary Figure S7). Subgroup analysis stratified by disease type indicated that the AECOPD subgroup results did not align with the overall results, warranting further validation through large-scale multicenter studies (Supplementary Figure S8). Sensitivity analysis confirmed the robustness and reliability of the conclusions (Supplementary Figure S11F).

#### 3.5.3 Imaging absorption rate

Three articles (Pan et al., 2020; Xie, 2017; Wei et al., 2016), involving 330 patients, reported on imaging absorption rate. The heterogeneity test indicated *P* = 0.30 and *I* ^*2*^ = 16%. Due to the low heterogeneity, a fixed-effects model was used. Compared with conventional biomedical therapy alone, Shufeng Jiedu Capsule combined with biomedical therapy may improve imaging absorption rates in respiratory diseases presenting with wind-heat syndrome (RR = 1.15, 95% CI 1.06 to 1.25, *P* = 0.0009) (Supplementary Figure S9). Sensitivity analysis confirmed the robustness and reliability of the conclusions (Supplementary Figure S11G).

#### 3.5.4 Adverse events

Sixteen articles (Li and Zhang, 2022; Zhang J. P., 2022; Chen and Zhang, 2021; Tang et al., 2021; Pan et al., 2020; Zhao and Wang, 2020; Wang and Zhao, 2020; Shen, 2021; Yang et al., 2019; He, 2019; Zhou et al., 2019; Wu et al., 2019; Zhu et al., 2018; Xie, 2017; Wei et al., 2016; Han et al., 2016), involving 1474 patients, reported adverse event incidence rates. The heterogeneity test indicated *P* = 0.80 and *I* ^*2*^ = 0%. As no heterogeneity was detected, a fixed-effects model was used. Compared with biomedical therapy alone, the combination of Shufeng Jiedu Capsule and biomedical therapy showed no statistically significant difference in adverse event incidence (RR = 0.88, 95% CI 0.34 to 2.31, *P* = 0.80). Sensitivity analysis confirmed the robustness and reliability of the conclusions (Supplementary Figure S11H). Subgroup analysis by respiratory disease type yielded results consistent with the overall meta-analysis (Supplementary Figure S10). Adverse events were mainly gastrointestinal, and all were mild, and no serious adverse events were reported. No studies reported any significant drug-drug interactions. Detailed descriptive statistics are presented in Table 2.

**TABLE 2:** Adverse events of included articles.

Study	Adverse events in the experimental group	Adverse events in the control group	System organ class	Severity
Zhao and Wang (2020)	Diarrhea (1 case) Proportion:1.28%	Mild nausea (2 cases), fatigue (1 case) Proportion:3.85%	Gastrointestinal and General Disorders	Mild
Wang and Zhang (2020)	Mild diarrhea (1 case) Proportion:2.04%	Vomiting (2 cases) Proportion:4.08%	Gastrointestinal	Mild
Xie (2017)	Not have Proportion:0%	Diarrhea (1 case) Proportion:2.22%	Gastrointestinal	Mild
Wei et al. (2016)	Nausea symptoms (2 cases) Proportion:3.33%	Abdominal pain (1 case) Proportion:1.67%	Gastrointestinal	Mild
Han et al. (2016)	Diarrhea (1 case), dry and bitter mouth (2 cases) Proportion:8.82%	Mild retching reaction (1 case), mild diarrhea (1 case) Proportion:5.88%	Gastrointestinal	Mild

### 3.6 Publication bias

Funnel plots were used to evaluate publication bias for outcomes with 10 or more studies, including clinical effective rate, CRP, and adverse events. The funnel plots for clinical effective rate, CRP, and adverse events appeared asymmetric, suggesting possible publication bias (Supplementary Figure S12). Egger’s test was then performed to assess its impact. The results indicated no significant publication bias for clinical effective rate (*P* = 0.179), CRP (*P* = 0.066), or adverse events (*P* = 0.208) (Supplementary Figure S13). Given the absence of statistical evidence for bias, we did not apply trim-and-fill adjustments to avoid overcorrection. Sensitivity analysis confirmed the robustness and reliability of the conclusions (Supplementary Figures S11A,E,H).

### 3.7 Quality of evidence

The GRADE approach was applied to assess the quality of evidence for 10 outcome indicators (Table 3). The results showed high-quality evidence for the effective rate of TCM syndromes, resolution time of cough, and resolution time of phlegm. Moderate-quality evidence was found for the resolution time of pulmonary rales, imaging absorption rate, and adverse events; low-quality evidence for the clinical effective rate and resolution time of fever; and very low-quality evidence for CRP and PCT. The main reasons for downgrading evidence quality included poor methodological quality of the included RCTs, substantial heterogeneity, small sample sizes, and potential publication bias.

**TABLE 3:** Summary of findings.

No.	Study design	Certainty assessment					Summary of results						Importance
		Risk of bias	Inconsistency	Indirectness	Imprecision	Other considerations	No of patients		Effect (95% CI)		Certainty		
							E	C	Relative	Absolute			
Clinical effective rate													
23	RCT	Serious^a^	not serious	not serious	not serious	Serious^b^	1249	1252	RR 1.16 (1.12–1.20)	126 more per 1,000 (from 95 more to 158 more)	⊕⊕○○	Low	Important
Effective rate of TCM syndromes													
7	RCT	not serious	not serious	not serious	not serious	not serious^g^	305	305	RR 1.17 (1.10–1.24)	136 more per 1,000 (from 80 more to 192 more)	⊕⊕⊕⊕	High	Important
Resolution time of fever													
6	RCT	Serious^a^	Serious^c^	not serious	not serious	not serious^g^	322	322	—	MD 1.03 lower (1.42 lower to 0.64 lower)	⊕⊕○○	Low	Important
Resolution time of cough													
6	RCT	not serious	not serious	not serious	not serious	not serious^g^	289	289	—	MD 0.97 lower (1.09 lower to 0.85 lower)	⊕⊕⊕⊕	High	Important
Resolution time of phlegm													
5	RCT	not serious	not serious	not serious	not serious	not serious^g^	236	236	—	MD 0.48 lower (0.79 lower to 0.17 lower)	⊕⊕⊕⊕	High	Important
Resolution time of pulmonary rales													
5	RCT	not serious	Serious^c^	not serious	not serious	not serious^g^	236	236	—	MD 0.83 lower (1.15 lower to 0.51 lower)	⊕⊕⊕○	Moderate	Important
CRP													
10	RCT	Serious^a^	very serious^d^	not serious	not serious	not serious^g^	552	552	—	SMD 0.99 lower (1.55 lower to 0.43 lower)	⊕○○○	Very low	Important
PCT													
5	RCT	very serious^e^	very serious^d^	not serious	not serious	not serious^g^	255	255	—	SMD 2.06 lower (3.62 lower to 0.49 lower)	⊕○○○	Very low	Important
Imaging absorption rate													
3	RCT	not serious	not serious	not serious	Serious^f^	not serious^g^	165	165	RR 1.15 (1.06–1.25)	123 more per 1,000 (from 49 more to 205 more)	⊕⊕⊕○	Moderate	Important
Adverse events													
16	RCT	not serious	not serious	not serious	not serious	Serious^b^	737	737	RR 0.88 (0.34–2.31)	1 fewer per 1,000 (from 7 fewer to 14 more)	⊕⊕⊕○	Moderate	Important

Abbreviations: RCT, randomized controlled trial; CI: confidence interval; MD: mean difference; RR: risk ratio; SMD: standardised mean difference.

^a^ Downgrade by one level: More than 25% of the studies were those with a higher risk of overall bias.

^b^ There was a risk of publication bias.

^c^ Downgrade by one level: Heterogeneity among the studies was fairly high.

^d^ Downgrade by two level: I^2^ ≥ 75% for heterogeneity, and neither subgroup analyses nor sensitivity analyses reduced its substantial heterogeneity.

^e^ Downgrade by two level: More than 50% of the studies were those with a higher risk of overall bias.

^f^ Downgrade by one level: The optimal information sample size was less than 400 participants.

^g^ No test for publication bias.

## 4 Discussion

Clinical syndrome patterns form the cornerstone of TCM diagnosis and treatment (Chen et al., 2022). The pattern-oriented disease classification framework organizes diseases as categories under pathogenic mechanisms and clinical patterns, which serve as guiding principles. This approach emphasizes pattern-targeted therapy and shows substantial overlap with the core concepts of precision medicine (Zhou et al., 2011). Wind-heat syndrome is a clinical pattern caused by the invasion of wind-heat pathogens. These pathogens impair the lung system’s defensive function, and the syndrome is primarily characterized by fever with aversion to wind, cough, and rhinorrhea. Classified as a disorder of the upper energizer, wind-heat syndrome is one of the most common patterns seen in respiratory diseases. Shufeng Jiedu Capsule, a CCPP, clears heat, resolves toxins, dispels wind-heat, and soothes the throat. It regulates the clinical manifestations of wind-heat syndrome by restoring the free flow of lung-defense qi, thereby expelling pathogenic wind-heat from the exterior, preventing deeper pathogenic invasion, and eliminating stagnant toxins. Shufeng Jiedu Capsule is, therefore, indicated for respiratory diseases with wind-heat syndrome. The National Medical Products Administration includes Shufeng Jiedu Capsule in several official guideline medication directories (National Medical Products Administration, 2014) for the treatment of lung infection–related diseases such as AECOPD, AECB, and CAP. Because CCPPs are multi-metabolite formulations, their pharmacological effects are the result of synergistic multi-target actions. This feature provides a practical pathway for precision medicine, making accurate clinical positioning essential to demonstrate therapeutic value (Yu et al., 2024). To this end, our study conducted a systematic review and meta-analysis of Shufeng Jiedu Capsule for the treatment of respiratory diseases with wind-heat syndrome, with the aim of providing evidence for precise clinical application and supporting the development of new syndrome-pattern–centered drugs.

Our analysis found that Shufeng Jiedu Capsule combined with conventional biomedical therapy was superior to biomedical therapy alone in improving the effective rate of TCM syndromes and in shortening the resolution time of cough and phlegm. These outcomes were supported by high-quality evidence according to the GRADE assessment. Improvements were also observed in imaging absorption rates and in the resolution time of pulmonary rales, although the certainty of evidence for these outcomes was moderate. In contrast, benefits for the clinical effective rate, resolution time of fever, and reductions in CRP and PCT levels were based on low or very low certainty evidence, indicating the need for confirmation in rigorously designed clinical trials. Subgroup analyses suggested that SFJD may provide benefits in AECOPD, CAP, acute upper respiratory tract infection (AURTI), and AECB. The therapeutic effects of SFJD are likely linked to its diverse pharmacological actions, which include antitussive, expectorant, anti-inflammatory, and immunomodulatory properties. Modern pharmacology supports these effects; for example, polydatin from the giant knotweed *Reynoutria japonica Houtt.* [*Polygonaceae; Polygoni Cuspidati Rhizoma et Radix*] in Shufeng Jiedu Capsule regulates inflammatory cytokine production (Lanzilli et al., 2012), while *Forsythia suspensa (Thunb.) Vahl* [*Oleaceae; Forsythiae Fructus*] and its active metabolite forsythoside exhibit antibacterial activity against *Streptococcus* pneumoniae and *Staphylococcus aureus* (Liu and Zhang, 2015). *Isatis indigotica subsp. tinctoria* [*Brassicaceae; Isatidis Radix*] demonstrates broad antibacterial effects, including activity against hemolytic streptococci, *Streptococcus* pneumoniae, and *Escherichia coli* (Gao, 2016), and *Bupleurum chinense DC.* [*Apiaceae; Bupleuri Radix*] with its saikosaponin A has antitussive effects, potentially through promoting adrenal corticosteroid synthesis (Wang et al., 2024). Additional contributions include *Verbena officinalis L.* [*Verbenaceae; Verbenae Herba*], which modulates immune function; *Patrinia scabiosifolia Link* [*Caprifoliaceae; Patriniae Herba*], which inhibits *Pseudomonas aeruginosa* and exhibits antiviral activity (Fu et al., 2017); *Phragmites australis (Cav.) Trin. ex Steud.* [*Poaceae; Phragmitis Rhizoma*], which improves microvascular permeability, controls vasodilation, reduces edema, and mitigates early inflammatory lesions (Li J. Z. et al., 2023); and *Glycyrrhiza uralensis Fisch. ex DC.* [*Fabaceae; Glycyrrhizae Radix Et Rhizoma*], which has antiviral effects and reduces inflammatory chemokine expression (Song et al., 2023). The combined pharmacological actions of Shufeng Jiedu Capsule include anti-inflammatory, antioxidant, pulmonary-function-enhancing, and immunomodulatory effects, mediated via multiple targets such as HRAS and MAP2K (Han et al., 2019).

Regarding safety, the current analysis indicated no significant difference between Shufeng Jiedu Capsules and the control groups. The reported adverse events were primarily mild, transient gastrointestinal symptoms (e.g., mild diarrhea, nausea, abdominal pain, mild retching), occurred infrequently, and resolved upon discontinuation of the treatment. No serious adverse events or significant drug-drug interactions were reported. However, the overall quality of safety reporting was low and inconsistent, preventing definitive conclusions. Future trials should prioritize standardized, comprehensive monitoring and clear reporting of all adverse events.

To explore the sources of heterogeneity in outcome measures with substantial variation, we conducted sensitivity analyses and subgroup analyses. Subgroup analyses were stratified by disease subtype, publication year, age, and treatment duration. Across all subgroups, the meta-analysis results were consistent with the overall pooled outcomes, although heterogeneity remained high. This persistent heterogeneity may be related to variations in study quality and inconsistency in outcome assessment criteria. Further sensitivity analyses indicated that clinical heterogeneity in the resolution time of fever and pulmonary rales might have originated from Wu Xia’s study (Wu et al., 2019), which exclusively enrolled female participants. This methodological deviation may explain the pronounced heterogeneity and instability of the results. Despite efforts to address heterogeneity, several outcomes continued to show considerable variation, necessitating cautious interpretation in clinical practice, particularly for the resolution time of fever, resolution time of pulmonary rales, and decreases in inflammatory markers such as CRP and PCT. Sensitivity analyses for other outcome indicators confirmed the robustness of the findings. Further investigation into unmeasured sources of heterogeneity identified three possible confounding factors: (1) Antibiotic co-intervention varied substantially across studies. For PCT outcomes, both groups in Wei et al. (Wei et al., 2016) received intravenous moxifloxacin alongside routine symptomatic treatments such as cough suppression and physical cooling, whereas Xia et al. (Xia et al., 2021) administered piperacillin–tazobactam. Other studies did not specify antibiotic regimens; for CRP outcomes, Yang et al. (Yang et al., 2019) and Li (Li, 2022) reported only basic supportive therapy, while the remaining studies used non-uniform antibiotics, including moxifloxacin, cefoperazone–sulbactam, piperacillin–tazobactam, and levofloxacin hydrochloride tablets. (2) The application and reporting of supportive symptomatic treatments were inconsistent across studies. (3) Baseline disease severity differed, particularly in PCT studies, where only Shen et al. (Shen H 2021) provided detailed baseline characteristics in tabular form; other studies simply stated that groups were comparable without reporting comprehensive baseline data. These factors may have contributed to the elevated heterogeneity observed. The core clinical value of this study lies in establishing syndrome-specific evidence for the efficacy of Shufeng Jiedu Capsule in treating wind-heat syndrome respiratory infections. Clinicians can optimize medication use according to the following principles: (1) Precision syndrome differentiation: Prioritize Shufeng Jiedu Capsule in patients who present with core wind-heat syndrome symptoms such as fever, sore throat with congestion, yellow sputum, and a red tongue with yellow coating. (2) Combination strategy: During acute exacerbation phases (e.g., AECOPD), combining Shufeng Jiedu Capsule with biomedicine (antivirals or antibiotics) can shorten symptom resolution time compared to biomedicine alone (Ren et al., 2024). (3) Treatment course control: Treatment duration should be adjusted based on changes in TCM syndrome scores; when the score decreases, reassessment of syndrome differentiation is warranted.

We assessed the quality of evidence for each outcome indicator to inform clinical practice. Risk of bias was evaluated using Cochrane criteria. The evidence was downgraded by one level if more than 25% of studies were at high risk of bias and by two levels if more than 50% were at high risk. Inconsistency was judged based on heterogeneity, with *I* ^*2*^ values between 50% and 75% prompting a one-level downgrade, and values ≥ 75% prompting a two-level downgrade. Although the resolution times for fever and pulmonary rales initially showed *I* ^2^ values ≥ 75%, heterogeneity decreased to 0% after subgroup analysis, leading to only a one-level downgrade. In contrast, CRP and PCT outcomes maintained *I* ^2^ values > 75% despite subgroup and sensitivity analyses, warranting two-level downgrades. No concerns regarding indirectness were identified based on the PICO framework. Imprecision was addressed by downgrading one level when the total sample size fell below the prespecified optimal information size of 400 participants. Publication bias was evaluated using funnel plot asymmetry and Egger’s test, with one-level downgrades applied to two outcomes where bias was suspected. In total, the final quality ratings were “high” for three outcome indicators, “moderate” for three, “low” for two, and “very low” for two. Funnel plot inspection suggested asymmetry, but Egger’s test did not detect statistically significant publication bias. It is important to note that funnel plot asymmetry can arise from causes other than publication bias, including heterogeneity, measurement error, or chance variation. Moreover, the limited number of studies reduced the statistical power to detect bias. Given the absence of statistical evidence for bias, we did not apply trim-and-fill adjustments to avoid overcorrection. Instead, we performed additional sensitivity analyses, which showed stable effect sizes after sequential exclusion of individual studies. The clinical recommendations from this review should be interpreted cautiously, given the low or very low quality of evidence for some outcomes. These limitations primarily stem from methodological weaknesses in the included trials, such as unclear allocation concealment and lack of blinding. Future high-quality RCTs, incorporating rigorous methods such as adequate blinding, proper allocation concealment, and prospective registration, are needed to provide more definitive guidance.

This study has several limitations: (1) A significant limitation of the present meta-analysis is the methodological quality of included studies. Risk of bias assessments revealed substantial shortcomings: none of the six studies clearly described random sequence generation methods, only one reported blinding, and none mentioned allocation concealment or blinding of outcome assessors. This extensive incompleteness in reporting crucial bias-control domains inherently limits the reliability of the evidence and may lead to inflated treatment effect estimates due to performance and selection biases. Although these deficiencies prevent definitive conclusions regarding efficacy, the consistent benefit direction across multiple studies suggests that the observed effects are unlikely attributable solely to bias. Nonetheless, the results must be interpreted with considerable caution, and future rigorous trials with detailed methodological transparency are urgently required. (2) This study focuses on respiratory diseases, and there may be clinical heterogeneity due to the inclusion of different disease types. (3) The included studies reported limited information on core TCM syndrome indicators, with only seven reporting the effectiveness rate of TCM syndromes using a unified reference standard. However, TCM syndrome scores could not be statistically analyzed because of the small number of reports and inconsistent reference standards. The inconsistent reference standards for TCM syndrome diagnosis in the included studies may also contribute to research heterogeneity. Only one study (Han et al., 2016) did not clearly report diagnostic criteria for TCM syndromes, potentially introducing some bias. However, the outcome measures it reported were exclusively limited to the low-heterogeneity outcomes pooled in this study (clinical effective rate and adverse events). Sensitivity analysis revealed the robustness and reliability of the conclusion; therefore, although this diagnostic omission may theoretically introduce selection bias, its impact was constrained by the restricted outcome reporting scope. (4) Differences in biomedical intervention measures among the included studies, along with uncontrolled variables such as patient age, gender, and other demographic factors, may also affect interpretation of the results. (5) Among the outcome indicators, clinical effectiveness rate and radiological absorption rate were analyzed only for studies explicitly reporting these measures, and the lack of a unified reference standard contributed to a certain degree of research heterogeneity. (6) The sample sizes of the included studies were not predetermined and varied considerably, which may lead to bias. (7) This study represents the first application of the GRADE system to evaluate the evidence quality of Shufeng Jiedu Capsule combination therapy. The results demonstrated high-quality evidence for outcomes such as the TCM syndrome response rate but also revealed the following key limitations: Impact of bias risk on effect sizes: Among the included RCTs, 96% failed to report implementation of blinding, which may have overestimated effect sizes for subjective outcomes, and sensitivity analysis showed reduced pooled RR after excluding relevant studies, whereas objective indicators such as CRP and PCT exhibited non-significant changes post-sensitivity analysis. Quantification of heterogeneity sources: Differences in assessment criteria for certain outcomes across research centers may have contributed to heterogeneity, requiring cautious interpretation. Clinical implications of evidence downgrades: Low-quality evidence indicators like CRP and PCT should also be interpreted with care, and future studies should adopt standardized detection protocols for inflammatory biomarkers. (8) Despite subgroup analyses, CRP and PCT results maintained substantial heterogeneity (*I* ^2^ = 89%), suggesting the influence of unmeasured confounders. These limitations may compromise the robustness of efficacy conclusions, particularly for patient-reported endpoints, and the findings should be verified by multicenter, high-quality, large-scale RCTs that reflect the characteristics and advantages of TCM.

Future Research Directions and Recommendations: (1) We recommend that future TCM clinical research should prioritize the standardization of syndrome differentiation criteria and employ a disease–syndrome combination model to improve research quality and establish a robust scientific foundation for the long-term development of TCM. Objective quantification of tongue and pulse characteristics using TCM four diagnostic instruments is encouraged to reduce subjectivity, while for diagnoses involving biomedicine, standardized diagnostic criteria should be applied consistently and supported by relevant imaging or pathogenic evidence. (2) Reporting criteria for TCM clinical outcome indicators should be standardized, with improvements in the reporting quality of efficacy evaluation measures and emphasis on TCM syndrome outcome indicators to facilitate quantitative assessment and research. (3) Basic research on proprietary Chinese medicines should be strengthened before market launch to avoid irrational clinical drug use, and post-marketing evaluations should be initiated early to develop a standardized, systematic, multi-dimensional, and multi-level clinical evaluation index system for such medicines (Liao et al., 2022). This would enable more objective and reliable results and promote the clinical value of these products. (4) Based on the disease–syndrome combination model, disease-oriented syndrome research should be complemented by the syndrome-oriented disease diagnosis and treatment model, in which TCM syndromes are the core. The current model of diagnosis and treatment is shifting toward greater precision for specific patients, diseases, and syndromes. Precision is a historical necessity for TCM development (He et al., 2020), and precise medication research based on syndrome-oriented disease models should be supported by master protocol designs similar to those used in precision medicine trials. This approach aligns with both the development patterns of clinical diseases and the dynamic evolution of syndromes, while emerging technologies can further advance precise clinical diagnosis and treatment models in TCM (Wang and Xie, 2020; Gan et al., 2021). Such developments will enhance the scientific rigor and efficacy advantages of TCM and accelerate modernization research (Chen et al., 2022). (5) Future studies should also investigate the long-term safety profile of Shufeng Jiedu Capsule, particularly in combination with other drugs, to enable more comprehensive safety evaluations that inform both clinical research and application. (6) Future trials should implement standardized assays for inflammatory biomarkers and explicitly correlate these objective measures with specific TCM syndrome patterns. This combined methodological approach will enhance scientific rigor and facilitate a meaningful integration of traditional applications with biomedical validation. (7) Finally, to generate more definitive clinical guidance, future rigorously conducted RCTs should implement essential methodologies including adequate blinding, allocation concealment, and prospective trial registration. This would prevent validity erosion stemming from low-certainty evidence.

## 5 Conclusion

Shufeng Jiedu Capsules combined with biomedicine may improve the clinical effective rate, effective rate of TCM syndromes, and imaging absorption rate. It may also shorten symptom resolution times for fever, cough, expectoration, and pulmonary rales, and reduce CRP and PCT levels. The adverse events were minor and controllable, and no serious adverse events were reported. However, these findings are based largely on low or very low certainty evidence, mainly due to methodological flaws in the included trials (notably, unclear allocation concealment, lack of blinding, and absence of protocol registration), necessitating cautious clinical interpretation. Multicenter RCTs with rigorous methods, including predefined allocation concealment, blinding, and prospective registration, are needed to confirm these results and to reflect the therapeutic characteristics of TCM syndrome differentiation.

## Data Availability

The original contributions presented in the study are included in the article/Supplementary Material, further inquiries can be directed to the corresponding author.
