# Supplementary material for: Shufeng Jiedu Capsules for treating wind-heat syndrome respiratory diseases: a systematic review and meta-analysis
Source: Front Pharmacol. 2025 Sep 18;16:1602563. doi: 10.3389/fphar.2025.1602563 (PMC12488728; doi:10.3389/fphar.2025.1602563)
Supplement: Supplementary file 1 [file DataSheet1.zip › Data Sheet 1 (1)/supplemental materials/Supplementary Appendix A2 File. PRISMA 2020 flow diagram.docx]

**Identification of studies via databases and registers**

Records identified from*:n=2047

Pubmed (n =62)

Embase (n =88)

Cochrane Library (n =21)

Web of Science (n= 74)

CNKI (n =488)

SinoMed (n =393)

Wanfang (n =487)

VIP (n=415)

Other sources (n =19)

Records removed *before screening*:

Duplicate records removed (n =1176)

**Identification**

Records excluded**

(n =797)

Records screened

(n =871)

Reports not retrieved

(n =3)

Reports sought for retrieval

(n =77)

**Screening**

Reports excluded: n =49

No confirmed wind-heat syndrome or patients not having respiratory diseases (n =19)

Not use SFJDC (n =3)

Not RCT (n =18)

Duplication (n =9)

Reports assessed for eligibility

(n =74)

Studies included in review

(n =25)

Reports of included studies

(n =25)

**Included**
