# Supplementary material for: Shufeng Jiedu Capsules for treating wind-heat syndrome respiratory diseases: a systematic review and meta-analysis
Source: Front Pharmacol. 2025 Sep 18;16:1602563. doi: 10.3389/fphar.2025.1602563 (PMC12488728; doi:10.3389/fphar.2025.1602563)
Supplement: Supplementary file 1 [file DataSheet1.zip › Data Sheet 1 (1)/supplemental materials/Supplementary Figures and Tables.docx]

Shufeng Jiedu capsules for treating wind-heat syndrome respiratory diseases: A systematic review and Meta-Analysis

Jia-Min Liu^1,2,3,4^, Lu Wang^1,2,3,4^, Gui-Xiang Zhao^1,2,3,4^, Hai-Long Zhang^1,2,3,4*^

^1^Department of Respiratory Diseases, the First Affiliated Hospital of Henan University of Chinese Medicine, Zhengzhou 450003, People’s Republic of China

^2^The First Clinical Medical College, Henan University of Chinese Medicine, Zhengzhou 450003, People’s Republic of China

^3^Collaborative Innovation Center for Chinese Medicine and Respiratory Diseases Co-Constructed by Henan Province & Education Ministry of P.R. China, Henan University of Chinese Medicine, 156 Jinshui East Road, Zhengzhou 450046, People’s Republic of China

^4^Henan Key Laboratory of Chinese Medicine for Respiratory Diseases, Henan University of Chinese Medicine, Zhengzhou 450046, People’s Republic of China

Keywords:

Shufeng Jiedu Capsules,

wind-heat syndrome,

respiratory diseases,

randomized controlled trials,

meta-analysis

*** Correspondence:** Hai-Long Zhang, Department of Respiratory Diseases, the First Affiliated Hospital of Henan University of Chinese Medicine, Zhengzhou 450003, People’s Republic of China
Corresponding Author email：zhanghailong6@126.com

Supplementary Material

**catalogue**

[Supplementary Material 2](#_Toc207355035)

[**catalogue** 2](#_Toc207355036)

[**Supplementary Table S1** Search strategies 4](#_Toc207355037)

[**Supplementary Table S2** Composition of Shufeng Jiedu Capsules 5](#_Toc207355038)

[**Supplementary Table S3:** Summary of composition characteristics of preparations in all included articles 6](#_Toc207355039)

[**Supplementary Figure S1.** Subgroup analysis of the Resolution time of fever. (A) Based on the study year; (B) Based on the mean age 18](#_Toc207355040)

[**Supplementary Figure S2.** Forest plot of Resolution time of fever before removal of Wu Xia's study 19](#_Toc207355041)

[**Supplementary Figure S3.** Subgroup analysis of the Resolution time of pulmonary rales. (A) Based on the study year, (B) Based on the mean age, (C) Based on the course of treatment 20](#_Toc207355042)

[**Supplementary Figure S4.** Forest plot of Resolution time of pulmonary rales before removal of Wu Xia's study 21](#_Toc207355043)

[**Supplementary Figure S5.** Subgroup analysis of CRP. (A) Based on the study year, (B) Based on the mean age, (C) Based on the course of treatment 22](#_Toc207355044)

[**Supplementary Figure S6.** Forest plot of CRP between experimental group and control group. 23](#_Toc207355045)

[**Supplementary Figure S7.** Subgroup analysis of PCT Based on the study year. 23](#_Toc207355046)

[**Supplementary Figure S8** Forest plot of PCT between experimental group and control group. 24](#_Toc207355047)

[**Supplementary Figure S9** Forest plot of Imaging absorption rate between experimental group and control group. 24](#_Toc207355048)

[**Supplementary Figure S10** Forest plot of Adverse Events between experimental group and control group. 25](#_Toc207355049)

[**Supplementary Figure S11.** Sensitivity analysis. (A) Clinical effective rate. (B) Effective rate of TCM syndromes. (C) Resolution time of cough (D) Resolution time of phlegm (E) CRP (F) PCT (G) Imaging absorption rate (H) Adverse Events 27](#_Toc207355050)

[**Supplementary Figure S12** Funnel plots of publication bias. (A) Clinical effective rate. (B) CRP (C) Adverse Events 28](#_Toc207355051)

[**Supplementary Figure S13** Egger’s plot. (A) Clinical effective rate. (B) CRP (C) Adverse Events 29](#_Toc207355052)

**Supplementary Table S1** Search strategies

| **Databases** | **Search terms** |
| --- | --- |
| Chinese search strategy | #1 疏风解毒胶囊 (Shufeng Jiedu Jiaonang) |
|  | #2 疏风解毒 (ShufengJiedu) |
|  | #3 #1 OR #2 |
| English search strategy | #1 Shufengjiedu |
|  | #2 Shu Feng Jie Du |
|  | #3 Shu-Feng-Jie-Du |
|  | #4 Shufeng-Jiedu |
|  | #5 Shufeng Jiedu |
|  | #6 SFJD |
|  | #7 Shu Feng Jie Du Capsules |
|  | #8 SFJDC |
|  | #9 #1 OR #2 OR #3 OR #4 OR #5 OR #6 OR #7 OR #8 |

**Supplementary Table S2** Composition of Shufeng Jiedu Capsules

| **No.** | **Latin Binomial (Authority)** | **Family** | **Pharmacopeial Drug Name** | **Part Used** |
| --- | --- | --- | --- | --- |
| 1 | *Bupleurum chinense DC.* | *Apiaceae* | *Bupleuri Radix* | Root |
| 2 | *Forsythia suspensa (Thunb.) Vahl* | *Oleaceae* | *Forsythiae Fructus* | Fruit |
| 3 | *Glycyrrhiza uralensis Fisch. ex DC.* | *Fabaceae* | *Glycyrrhizae Radix Et Rhizoma* | Root & Rhizome |
| 4 | *Isatis indigotica subsp. tinctoria* | *Brassicaceae* | *Isatidis Radix* | Root |
| 5 | *Patrinia scabiosifolia Link* | *Caprifoliaceae* | *Patriniae Herba* | Aerial parts |
| 6 | *Phragmites australis (Cav.) Trin. ex Steud.* | *Poaceae* | *Phragmitis Rhizoma* | Rhizome |
| 7 | *Reynoutria japonica Houtt.* | *Polygonaceae* | *Polygoni Cuspidati Rhizoma et Radix* | Rhizome & Root |
| 8 | *Verbena officinalis L.* | *Verbenaceae* | *Verbenae Herba* | Aerial parts |

**Supplementary Table S3:** Summary of composition characteristics of preparations in all included articles

| **Study ID** | **Formulation** | **Source** | **Species, concentration** | **Quality control reported?**  **(Y/N)** | **Approval Number? (Y/N)** | **Batch Number? (Y/N)** | **Chemical analysis reported?**  **(Y/N)** |
| --- | --- | --- | --- | --- | --- | --- | --- |
| Li YB 2023 | Shufeng Jiedu Capsules | Anhui Jiren Pharmaceutical Co., Ltd. | *Bupleurum chinense DC. [Apiaceae; Bupleuri Radix],* *Forsythia suspensa (Thunb.) Vahl [Oleaceae; Forsythiae Fructus],* *Glycyrrhiza uralensis Fisch. ex DC. [Fabaceae; Glycyrrhizae Radix Et Rhizoma],* *Isatis indigotica subsp. tinctoria [Brassicaceae; Isatidis Radix],* *Patrinia scabiosifolia Link [Caprifoliaceae; Patriniae Herba],* *Phragmites australis (Cav.) Trin. ex Steud. [Poaceae; Phragmitis Rhizoma],* *Reynoutria japonica Houtt. [Polygonaceae; Polygoni Cuspidati Rhizoma et Radix],* *Verbena officinalis L. [Verbenaceae; Verbenae Herba]* ；  0.52g/capsule | Y-Prepared according to Pharmacopoeia of the People's Republic of China (2020 Edition, Volume I) and National Medical Products Administration Standards YBZ05182019. | Y-National Drug Approval Number Z20090047 | Y-3201215 | Y-*Reynoutria japonica Houtt. [Polygonaceae; Polygoni Cuspidati Rhizoma et Radix]* contains polydatin as its active constituent, which effectively attenuates airway inflammation in asthmatic mice and reduces oxidative stress by mitigating inflammatory responses;  The primary bioactive components of *Forsythia suspensa (Thunb.) Vahl [Oleaceae; Forsythiae Fructus]* include lignans, terpenoids, phenolic acids, and volatile oils, demonstrating pharmacological effects such as anti-inflammatory, antiviral, hepatoprotective, and antioxidant activities. |
| Li M 2022 | Shufeng Jiedu Capsules | N | *Bupleurum chinense DC. [Apiaceae; Bupleuri Radix], Forsythia suspensa (Thunb.) Vahl [Oleaceae; Forsythiae Fructus], Glycyrrhiza uralensis Fisch. ex DC. [Fabaceae; Glycyrrhizae Radix Et Rhizoma], Isatis indigotica subsp. tinctoria [Brassicaceae; Isatidis Radix], Patrinia scabiosifolia Link [Caprifoliaceae; Patriniae Herba], Phragmites australis (Cav.) Trin. ex Steud. [Poaceae; Phragmitis Rhizoma], Reynoutria japonica Houtt. [Polygonaceae; Polygoni Cuspidati Rhizoma et Radix], Verbena officinalis L. [Verbenaceae; Verbenae Herba]* ；  0.52g/capsule | N | N | N | N |
| Li YB 2022 | Shufeng Jiedu Capsules | Anhui Jiren Pharmaceutical Co., Ltd. | *Bupleurum chinense DC. [Apiaceae; Bupleuri Radix], Forsythia suspensa (Thunb.) Vahl [Oleaceae; Forsythiae Fructus], Glycyrrhiza uralensis Fisch. ex DC. [Fabaceae; Glycyrrhizae Radix Et Rhizoma], Isatis indigotica subsp. tinctoria [Brassicaceae; Isatidis Radix], Patrinia scabiosifolia Link [Caprifoliaceae; Patriniae Herba], Phragmites australis (Cav.) Trin. ex Steud. [Poaceae; Phragmitis Rhizoma], Reynoutria japonica Houtt. [Polygonaceae; Polygoni Cuspidati Rhizoma et Radix], Verbena officinalis L. [Verbenaceae; Verbenae Herba]* ；  0.52g/capsule | Y-Prepared according to Pharmacopoeia of the People's Republic of China (2020 Edition, Volume I) and National Medical Products Administration Standards YBZ05182019. | Y-National Drug Approval Number Z20090047 | N | Y-Forsythin from *Forsythia suspensa (Thunb.) Vahl [Oleaceae; Forsythiae Fructus]* demonstrates anti-infection effects by enhancing immune cell differentiation, improving immunity, and reducing inflammatory cytokine levels. |
| Zhang JP 2022 | Shufeng Jiedu Capsules | Anhui Jiren Pharmaceutical Co., Ltd. | *Bupleurum chinense DC. [Apiaceae; Bupleuri Radix], Forsythia suspensa (Thunb.) Vahl [Oleaceae; Forsythiae Fructus], Glycyrrhiza uralensis Fisch. ex DC. [Fabaceae; Glycyrrhizae Radix Et Rhizoma], Isatis indigotica subsp. tinctoria [Brassicaceae; Isatidis Radix], Patrinia scabiosifolia Link [Caprifoliaceae; Patriniae Herba], Phragmites australis (Cav.) Trin. ex Steud. [Poaceae; Phragmitis Rhizoma], Reynoutria japonica Houtt. [Polygonaceae; Polygoni Cuspidati Rhizoma et Radix], Verbena officinalis L. [Verbenaceae; Verbenae Herba]* ；  0.52g/capsule | N | N | N | N |
| Chen W 2021 | Shufeng Jiedu Capsules | Anhui Jiren Pharmaceutical Co., Ltd. | *Bupleurum chinense DC. [Apiaceae; Bupleuri Radix], Forsythia suspensa (Thunb.) Vahl [Oleaceae; Forsythiae Fructus], Glycyrrhiza uralensis Fisch. ex DC. [Fabaceae; Glycyrrhizae Radix Et Rhizoma], Isatis indigotica subsp. tinctoria [Brassicaceae; Isatidis Radix], Patrinia scabiosifolia Link [Caprifoliaceae; Patriniae Herba], Phragmites australis (Cav.) Trin. ex Steud. [Poaceae; Phragmitis Rhizoma], Reynoutria japonica Houtt. [Polygonaceae; Polygoni Cuspidati Rhizoma et Radix], Verbena officinalis L. [Verbenaceae; Verbenae Herba]* ；  0.52g/capsule | Y- prepared according to Pharmacopedia of the People's Republic of China (2015) and National Medical Products Administration Standards  YBZ05182019 | N | Y-130601 | N |
| Tang ZQ 2021 | Shufeng Jiedu Capsules | Anhui Jiren Pharmaceutical Co., Ltd. | *Bupleurum chinense DC. [Apiaceae; Bupleuri Radix], Forsythia suspensa (Thunb.) Vahl [Oleaceae; Forsythiae Fructus], Glycyrrhiza uralensis Fisch. ex DC. [Fabaceae; Glycyrrhizae Radix Et Rhizoma], Isatis indigotica subsp. tinctoria [Brassicaceae; Isatidis Radix], Patrinia scabiosifolia Link [Caprifoliaceae; Patriniae Herba], Phragmites australis (Cav.) Trin. ex Steud. [Poaceae; Phragmitis Rhizoma], Reynoutria japonica Houtt. [Polygonaceae; Polygoni Cuspidati Rhizoma et Radix], Verbena officinalis L. [Verbenaceae; Verbenae Herba]* ；  0.52g/capsule | Y- prepared according to Pharmacopedia of the People's Republic of China (2015) and National Medical Products Administration Standards  YBZ05182019 | Y-National Drug Approval Number Z20090047 | N | N |
| Xia J 2021 | Shufeng Jiedu Capsules | Anhui Jiren Pharmaceutical Co., Ltd. | *Bupleurum chinense DC. [Apiaceae; Bupleuri Radix], Forsythia suspensa (Thunb.) Vahl [Oleaceae; Forsythiae Fructus], Glycyrrhiza uralensis Fisch. ex DC. [Fabaceae; Glycyrrhizae Radix Et Rhizoma], Isatis indigotica subsp. tinctoria [Brassicaceae; Isatidis Radix], Patrinia scabiosifolia Link [Caprifoliaceae; Patriniae Herba], Phragmites australis (Cav.) Trin. ex Steud. [Poaceae; Phragmitis Rhizoma], Reynoutria japonica Houtt. [Polygonaceae; Polygoni Cuspidati Rhizoma et Radix], Verbena officinalis L. [Verbenaceae; Verbenae Herba]* ；  0.52g/capsule | Y- prepared according to Pharmacopedia of the People's Republic of China (2015) and National Medical Products Administration Standards  YBZ05182019 | N | Y-3190512 | N |
| Shen H 2021 | Shufeng Jiedu Capsules | Anhui Jiren Pharmaceutical Co., Ltd. | *Bupleurum chinense DC. [Apiaceae; Bupleuri Radix], Forsythia suspensa (Thunb.) Vahl [Oleaceae; Forsythiae Fructus], Glycyrrhiza uralensis Fisch. ex DC. [Fabaceae; Glycyrrhizae Radix Et Rhizoma], Isatis indigotica subsp. tinctoria [Brassicaceae; Isatidis Radix], Patrinia scabiosifolia Link [Caprifoliaceae; Patriniae Herba], Phragmites australis (Cav.) Trin. ex Steud. [Poaceae; Phragmitis Rhizoma], Reynoutria japonica Houtt. [Polygonaceae; Polygoni Cuspidati Rhizoma et Radix], Verbena officinalis L. [Verbenaceae; Verbenae Herba]* ；  0.52g/capsule | Y- prepared according to Pharmacopedia of the People's Republic of China (2015) and National Medical Products Administration Standards  YBZ05182019 | Y-National Drug Approval Number Z200900470 | N | Y-Polydatin (resveratrol glucoside) from *Reynoutria japonica Houtt. [Polygonaceae; Polygoni Cuspidati Rhizoma et Radix]* exhibits antitussive and anti-asthmatic effects, while also regulating cardiovascular and gastrointestinal functions. It demonstrates additional pharmacological activities including antitumor, hepatoprotective, and choleretic effects;  Forsythoside from *Forsythia suspensa (Thunb.) Vahl [Oleaceae; Forsythiae Fructus]* possesses antiviral, anti-inflammatory, and antipyretic properties;  Bioalkaloids and organic acids in *Isatis indigotica subsp. tinctoria [Brassicaceae; Isatidis Radix]* are associated with antiviral activity;  Saponins and volatile oils in *Bupleurum chinense DC. [Apiaceae; Bupleuri Radix]* contribute to antipyretic and anti-inflammatory functions by inhibiting leukocyte migration, suppressing inflammatory mediator processes, reducing capillary permeability, and exhibiting antiviral, immunoenhancing, hepatoprotective, and lipid-lowering effects;  Verbenalin from *Verbena officinalis L. [Verbenaceae; Verbenae Herba]* demonstrates antibacterial, antiviral, and immune-enhancing properties;  *Glycyrrhiza uralensis Fisch. ex DC. [Fabaceae; Glycyrrhizae Radix Et Rhizoma]* primarily contains glycyrrhizin and glycyrrhetinic acid. Glycyrrhetinic acid inhibits cellular damage and exerts antioxidant effects while alleviating cough and phlegm symptoms. Glycyrrhizin and glycyrrhizic acid achieve anti-inflammatory, antibacterial, antiviral, and anti-allergic effects by blocking the production and release of inflammatory mediators such as macrophages. |
| Fei L 2020 | Shufeng Jiedu Capsules | Anhui Jiren Pharmaceutical Co., Ltd. | *Bupleurum chinense DC. [Apiaceae; Bupleuri Radix], Forsythia suspensa (Thunb.) Vahl [Oleaceae; Forsythiae Fructus], Glycyrrhiza uralensis Fisch. ex DC. [Fabaceae; Glycyrrhizae Radix Et Rhizoma], Isatis indigotica subsp. tinctoria [Brassicaceae; Isatidis Radix], Patrinia scabiosifolia Link [Caprifoliaceae; Patriniae Herba], Phragmites australis (Cav.) Trin. ex Steud. [Poaceae; Phragmitis Rhizoma], Reynoutria japonica Houtt. [Polygonaceae; Polygoni Cuspidati Rhizoma et Radix], Verbena officinalis L. [Verbenaceae; Verbenae Herba]* ；  0.52g/capsule | Y- prepared according to Pharmacopedia of the People's Republic of China (2015) and National Medical Products Administration Standards  YBZ05182019 | Y-National Drug Approval Number Z20090047 | N | N |
| Pan KL 2020 | Shufeng Jiedu Capsules | Anhui Jiren Pharmaceutical Co., Ltd. | *Bupleurum chinense DC. [Apiaceae; Bupleuri Radix], Forsythia suspensa (Thunb.) Vahl [Oleaceae; Forsythiae Fructus], Glycyrrhiza uralensis Fisch. ex DC. [Fabaceae; Glycyrrhizae Radix Et Rhizoma], Isatis indigotica subsp. tinctoria [Brassicaceae; Isatidis Radix], Patrinia scabiosifolia Link [Caprifoliaceae; Patriniae Herba], Phragmites australis (Cav.) Trin. ex Steud. [Poaceae; Phragmitis Rhizoma], Reynoutria japonica Houtt. [Polygonaceae; Polygoni Cuspidati Rhizoma et Radix], Verbena officinalis L. [Verbenaceae; Verbenae Herba]* ；  0.52g/capsule | N | N | N | N |
| Zhang B 2020 | Shufeng Jiedu Capsules | Anhui Jiren Pharmaceutical Co., Ltd. | *Bupleurum chinense DC. [Apiaceae; Bupleuri Radix], Forsythia suspensa (Thunb.) Vahl [Oleaceae; Forsythiae Fructus], Glycyrrhiza uralensis Fisch. ex DC. [Fabaceae; Glycyrrhizae Radix Et Rhizoma], Isatis indigotica subsp. tinctoria [Brassicaceae; Isatidis Radix], Patrinia scabiosifolia Link [Caprifoliaceae; Patriniae Herba], Phragmites australis (Cav.) Trin. ex Steud. [Poaceae; Phragmitis Rhizoma], Reynoutria japonica Houtt. [Polygonaceae; Polygoni Cuspidati Rhizoma et Radix], Verbena officinalis L. [Verbenaceae; Verbenae Herba]* ；  0.52g/capsule | Y- prepared according to Pharmacopedia of the People's Republic of China (2015) and National Medical Products Administration Standards YBZ05182019 | Y-National Drug Approval Number Z20090047 | N | N |
| Zhao LB 2020 | Shufeng Jiedu Capsules | Anhui Jiren Pharmaceutical Co., Ltd. | *Bupleurum chinense DC. [Apiaceae; Bupleuri Radix], Forsythia suspensa (Thunb.) Vahl [Oleaceae; Forsythiae Fructus], Glycyrrhiza uralensis Fisch. ex DC. [Fabaceae; Glycyrrhizae Radix Et Rhizoma], Isatis indigotica subsp. tinctoria [Brassicaceae; Isatidis Radix], Patrinia scabiosifolia Link [Caprifoliaceae; Patriniae Herba], Phragmites australis (Cav.) Trin. ex Steud. [Poaceae; Phragmitis Rhizoma], Reynoutria japonica Houtt. [Polygonaceae; Polygoni Cuspidati Rhizoma et Radix], Verbena officinalis L. [Verbenaceae; Verbenae Herba]* ；  0.52g/capsule | Y- prepared according to Pharmacopedia of the People's Republic of China (2015) and National Medical Products Administration Standards YBZ05182019 | Y-National Drug Approval Number Z20090047 | N | N |
| Wang ZB 2020 | Shufeng Jiedu Capsules | Anhui Jiren Pharmaceutical Co., Ltd. | *Bupleurum chinense DC. [Apiaceae; Bupleuri Radix], Forsythia suspensa (Thunb.) Vahl [Oleaceae; Forsythiae Fructus], Glycyrrhiza uralensis Fisch. ex DC. [Fabaceae; Glycyrrhizae Radix Et Rhizoma], Isatis indigotica subsp. tinctoria [Brassicaceae; Isatidis Radix], Patrinia scabiosifolia Link [Caprifoliaceae; Patriniae Herba], Phragmites australis (Cav.) Trin. ex Steud. [Poaceae; Phragmitis Rhizoma], Reynoutria japonica Houtt. [Polygonaceae; Polygoni Cuspidati Rhizoma et Radix], Verbena officinalis L. [Verbenaceae; Verbenae Herba]* ；  0.52g/capsule | Y- prepared according to Pharmacopedia of the People's Republic of China (2015) and National Medical Products Administration Standards | Y-National Drug Approval Number Z20090047 | N | N |
| Yang TW 2019 | Shufeng Jiedu Capsules | Anhui Jiren Pharmaceutical Co., Ltd. | *Bupleurum chinense DC. [Apiaceae; Bupleuri Radix], Forsythia suspensa (Thunb.) Vahl [Oleaceae; Forsythiae Fructus], Glycyrrhiza uralensis Fisch. ex DC. [Fabaceae; Glycyrrhizae Radix Et Rhizoma], Isatis indigotica subsp. tinctoria [Brassicaceae; Isatidis Radix], Patrinia scabiosifolia Link [Caprifoliaceae; Patriniae Herba], Phragmites australis (Cav.) Trin. ex Steud. [Poaceae; Phragmitis Rhizoma], Reynoutria japonica Houtt. [Polygonaceae; Polygoni Cuspidati Rhizoma et Radix], Verbena officinalis L. [Verbenaceae; Verbenae Herba]* ；  0.52g/capsule | Y- prepared according to Pharmacopedia of the People's Republic of China (2015) and National Medical Products Administration Standards | N | Y-20170512 | N |
| He J 2019 | Shufeng Jiedu Capsules | Anhui Jiren Pharmaceutical Co., Ltd. | *Bupleurum chinense DC. [Apiaceae; Bupleuri Radix], Forsythia suspensa (Thunb.) Vahl [Oleaceae; Forsythiae Fructus], Glycyrrhiza uralensis Fisch. ex DC. [Fabaceae; Glycyrrhizae Radix Et Rhizoma], Isatis indigotica subsp. tinctoria [Brassicaceae; Isatidis Radix], Patrinia scabiosifolia Link [Caprifoliaceae; Patriniae Herba], Phragmites australis (Cav.) Trin. ex Steud. [Poaceae; Phragmitis Rhizoma], Reynoutria japonica Houtt. [Polygonaceae; Polygoni Cuspidati Rhizoma et Radix], Verbena officinalis L. [Verbenaceae; Verbenae Herba]* ；  0.52g/capsule | Y- prepared according to Pharmacopedia of the People's Republic of China (2015) and National Medical Products Administration Standards | Y- National Drug Approval Number Z20090047 | Y-130601 | Y-Polydatin from *Reynoutria japonica Houtt. [Polygonaceae; Polygoni Cuspidati Rhizoma et Radix]* has been demonstrated to regulate the production of inflammatory cytokines both in vivo and in vitro, and reduces IL-17 production in monocytes by decreasing IL-17 mRNA levels;  Forsythoside A from *Forsythia suspensa (Thunb.) Vahl [Oleaceae; Forsythiae Fructus]* is highly likely to be the primary anti-inflammatory active component;  Saikosaponin A and other saponins in *Bupleurum chinense DC. [Apiaceae; Bupleuri Radix]* exhibit potent antitussive effects;  Glycyrrhizin from *Glycyrrhiza uralensis Fisch. ex DC. [Fabaceae; Glycyrrhizae Radix Et Rhizoma]* reduces the expression of inflammatory chemokines and pro-inflammatory factors through peroxidase activity, mitigates lipid protein denaturation and ovalbumin damage induced by reactive oxygen species (ROS), and alleviates clinical symptoms. |
| Zhou WB 2019 | Shufeng Jiedu Capsules | Anhui Jiren Pharmaceutical Co., Ltd. | *Bupleurum chinense DC. [Apiaceae; Bupleuri Radix], Forsythia suspensa (Thunb.) Vahl [Oleaceae; Forsythiae Fructus], Glycyrrhiza uralensis Fisch. ex DC. [Fabaceae; Glycyrrhizae Radix Et Rhizoma], Isatis indigotica subsp. tinctoria [Brassicaceae; Isatidis Radix], Patrinia scabiosifolia Link [Caprifoliaceae; Patriniae Herba], Phragmites australis (Cav.) Trin. ex Steud. [Poaceae; Phragmitis Rhizoma], Reynoutria japonica Houtt. [Polygonaceae; Polygoni Cuspidati Rhizoma et Radix], Verbena officinalis L. [Verbenaceae; Verbenae Herba]* ；  0.52g/capsule | Y- prepared according to Pharmacopedia of the People's Republic of China (2015) and National Medical Products Administration Standards | Y- National Drug Approval Number Z20090047 | N | N |
| Wu X 2019 | Shufeng Jiedu Capsules | Anhui Jiren Pharmaceutical Co., Ltd. | *Bupleurum chinense DC. [Apiaceae; Bupleuri Radix], Forsythia suspensa (Thunb.) Vahl [Oleaceae; Forsythiae Fructus], Glycyrrhiza uralensis Fisch. ex DC. [Fabaceae; Glycyrrhizae Radix Et Rhizoma], Isatis indigotica subsp. tinctoria [Brassicaceae; Isatidis Radix], Patrinia scabiosifolia Link [Caprifoliaceae; Patriniae Herba], Phragmites australis (Cav.) Trin. ex Steud. [Poaceae; Phragmitis Rhizoma], Reynoutria japonica Houtt. [Polygonaceae; Polygoni Cuspidati Rhizoma et Radix], Verbena officinalis L. [Verbenaceae; Verbenae Herba]* ；  0.52g/capsule | Y- prepared according to Pharmacopedia of the People's Republic of China (2015) and National Medical Products Administration Standards | N | Y-20160117，  20170325 | N |
| Chang Q 2019 | Shufeng Jiedu Capsules | Anhui Jiren Pharmaceutical Co., Ltd. | *Bupleurum chinense DC. [Apiaceae; Bupleuri Radix], Forsythia suspensa (Thunb.) Vahl [Oleaceae; Forsythiae Fructus], Glycyrrhiza uralensis Fisch. ex DC. [Fabaceae; Glycyrrhizae Radix Et Rhizoma], Isatis indigotica subsp. tinctoria [Brassicaceae; Isatidis Radix], Patrinia scabiosifolia Link [Caprifoliaceae; Patriniae Herba], Phragmites australis (Cav.) Trin. ex Steud. [Poaceae; Phragmitis Rhizoma], Reynoutria japonica Houtt. [Polygonaceae; Polygoni Cuspidati Rhizoma et Radix], Verbena officinalis L. [Verbenaceae; Verbenae Herba]* ；  0.52g/capsule | Y- prepared according to Pharmacopedia of the People's Republic of China (2015) and National Medical Products Administration Standards | Y- National Drug Approval Number Z20090047 | N | N |
| Li HP 2019 | Shufeng Jiedu Capsules | Anhui Jiren Pharmaceutical Co., Ltd. | *Bupleurum chinense DC. [Apiaceae; Bupleuri Radix], Forsythia suspensa (Thunb.) Vahl [Oleaceae; Forsythiae Fructus], Glycyrrhiza uralensis Fisch. ex DC. [Fabaceae; Glycyrrhizae Radix Et Rhizoma], Isatis indigotica subsp. tinctoria [Brassicaceae; Isatidis Radix], Patrinia scabiosifolia Link [Caprifoliaceae; Patriniae Herba], Phragmites australis (Cav.) Trin. ex Steud. [Poaceae; Phragmitis Rhizoma], Reynoutria japonica Houtt. [Polygonaceae; Polygoni Cuspidati Rhizoma et Radix], Verbena officinalis L. [Verbenaceae; Verbenae Herba]* ；  0.52g/capsule | N | N | N | N |
| Zhu CD 2018 | Shufeng Jiedu Capsules | Anhui Jiren Pharmaceutical Co., Ltd. | *Bupleurum chinense DC. [Apiaceae; Bupleuri Radix], Forsythia suspensa (Thunb.) Vahl [Oleaceae; Forsythiae Fructus], Glycyrrhiza uralensis Fisch. ex DC. [Fabaceae; Glycyrrhizae Radix Et Rhizoma], Isatis indigotica subsp. tinctoria [Brassicaceae; Isatidis Radix], Patrinia scabiosifolia Link [Caprifoliaceae; Patriniae Herba], Phragmites australis (Cav.) Trin. ex Steud. [Poaceae; Phragmitis Rhizoma], Reynoutria japonica Houtt. [Polygonaceae; Polygoni Cuspidati Rhizoma et Radix], Verbena officinalis L. [Verbenaceae; Verbenae Herba]* ；  0.52g/capsule | Y- prepared according to National Medical Products Administration Standards | Y- National Drug Approval Number Z20090047 | N | N |
| Zhao JL 2018 | Shufeng Jiedu Capsules | Anhui Jiren Pharmaceutical Co., Ltd. | *Bupleurum chinense DC. [Apiaceae; Bupleuri Radix], Forsythia suspensa (Thunb.) Vahl [Oleaceae; Forsythiae Fructus], Glycyrrhiza uralensis Fisch. ex DC. [Fabaceae; Glycyrrhizae Radix Et Rhizoma], Isatis indigotica subsp. tinctoria [Brassicaceae; Isatidis Radix], Patrinia scabiosifolia Link [Caprifoliaceae; Patriniae Herba], Phragmites australis (Cav.) Trin. ex Steud. [Poaceae; Phragmitis Rhizoma], Reynoutria japonica Houtt. [Polygonaceae; Polygoni Cuspidati Rhizoma et Radix], Verbena officinalis L. [Verbenaceae; Verbenae Herba]* ；  0.52g/capsule | Y- prepared according to National Medical Products Administration Standards | Y- National Drug Approval Number Z20090047 | N | N |
| Xie J 2017 | Shufeng Jiedu Capsules | Anhui Jiren Pharmaceutical Co., Ltd. | *Bupleurum chinense DC. [Apiaceae; Bupleuri Radix], Forsythia suspensa (Thunb.) Vahl [Oleaceae; Forsythiae Fructus], Glycyrrhiza uralensis Fisch. ex DC. [Fabaceae; Glycyrrhizae Radix Et Rhizoma], Isatis indigotica subsp. tinctoria [Brassicaceae; Isatidis Radix], Patrinia scabiosifolia Link [Caprifoliaceae; Patriniae Herba], Phragmites australis (Cav.) Trin. ex Steud. [Poaceae; Phragmitis Rhizoma], Reynoutria japonica Houtt. [Polygonaceae; Polygoni Cuspidati Rhizoma et Radix], Verbena officinalis L. [Verbenaceae; Verbenae Herba]* ；  0.52g/capsule | Y- prepared according to National Medical Products Administration Standards | Y- National Drug Approval Number Z20090047 | N | N |
| Wei B 2016 | Shufeng Jiedu Capsules | Anhui Jiren Pharmaceutical Co., Ltd. | *Bupleurum chinense DC. [Apiaceae; Bupleuri Radix], Forsythia suspensa (Thunb.) Vahl [Oleaceae; Forsythiae Fructus], Glycyrrhiza uralensis Fisch. ex DC. [Fabaceae; Glycyrrhizae Radix Et Rhizoma], Isatis indigotica subsp. tinctoria [Brassicaceae; Isatidis Radix], Patrinia scabiosifolia Link [Caprifoliaceae; Patriniae Herba], Phragmites australis (Cav.) Trin. ex Steud. [Poaceae; Phragmitis Rhizoma], Reynoutria japonica Houtt. [Polygonaceae; Polygoni Cuspidati Rhizoma et Radix], Verbena officinalis L. [Verbenaceae; Verbenae Herba]* ；  0.52g/capsule | N | N | N | N |
| Han YH 2016 | Shufeng Jiedu Capsules | Anhui Jiren Pharmaceutical Co., Ltd. | *Bupleurum chinense DC. [Apiaceae; Bupleuri Radix], Forsythia suspensa (Thunb.) Vahl [Oleaceae; Forsythiae Fructus], Glycyrrhiza uralensis Fisch. ex DC. [Fabaceae; Glycyrrhizae Radix Et Rhizoma], Isatis indigotica subsp. tinctoria [Brassicaceae; Isatidis Radix], Patrinia scabiosifolia Link [Caprifoliaceae; Patriniae Herba], Phragmites australis (Cav.) Trin. ex Steud. [Poaceae; Phragmitis Rhizoma], Reynoutria japonica Houtt. [Polygonaceae; Polygoni Cuspidati Rhizoma et Radix], Verbena officinalis L. [Verbenaceae; Verbenae Herba]* ；  0.52g/capsule | Y- prepared according to National Medical Products Administration Standards | Y- National Drug Approval Number Z20090047 | Y-100533 | N |
| Zhang YP 2014 | Shufeng Jiedu Capsules | Anhui Jiren Pharmaceutical Co., Ltd. | *Bupleurum chinense DC. [Apiaceae; Bupleuri Radix], Forsythia suspensa (Thunb.) Vahl [Oleaceae; Forsythiae Fructus], Glycyrrhiza uralensis Fisch. ex DC. [Fabaceae; Glycyrrhizae Radix Et Rhizoma], Isatis indigotica subsp. tinctoria [Brassicaceae; Isatidis Radix], Patrinia scabiosifolia Link [Caprifoliaceae; Patriniae Herba], Phragmites australis (Cav.) Trin. ex Steud. [Poaceae; Phragmitis Rhizoma], Reynoutria japonica Houtt. [Polygonaceae; Polygoni Cuspidati Rhizoma et Radix], Verbena officinalis L. [Verbenaceae; Verbenae Herba]* ；  0.52g/capsule | Y- prepared according to National Medical Products Administration Standards | Y- National Drug Approval Number Z20090047 | Y-130601 | N |

**Notes:** Concentration of each species in the preparations belong to the core technology of the pharaceuical companies, so they did not report the grams of each composition.

(A)
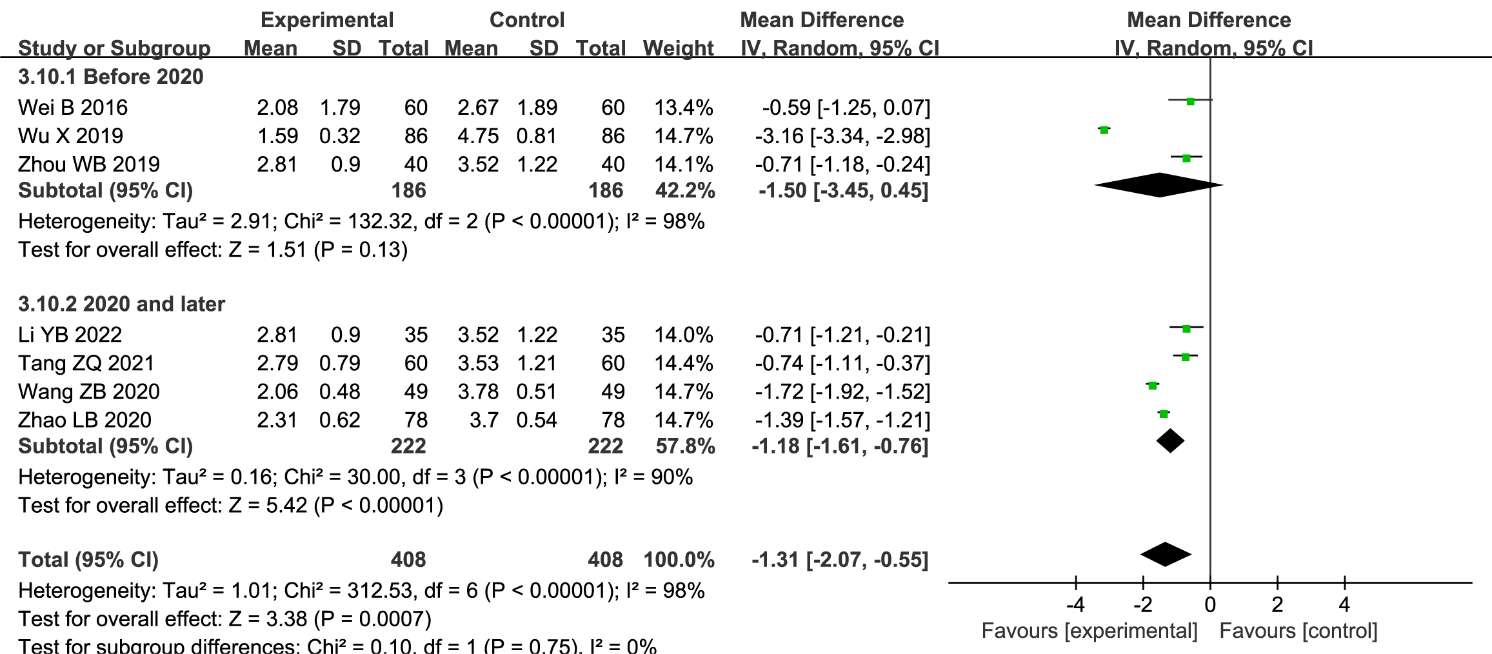


(B)
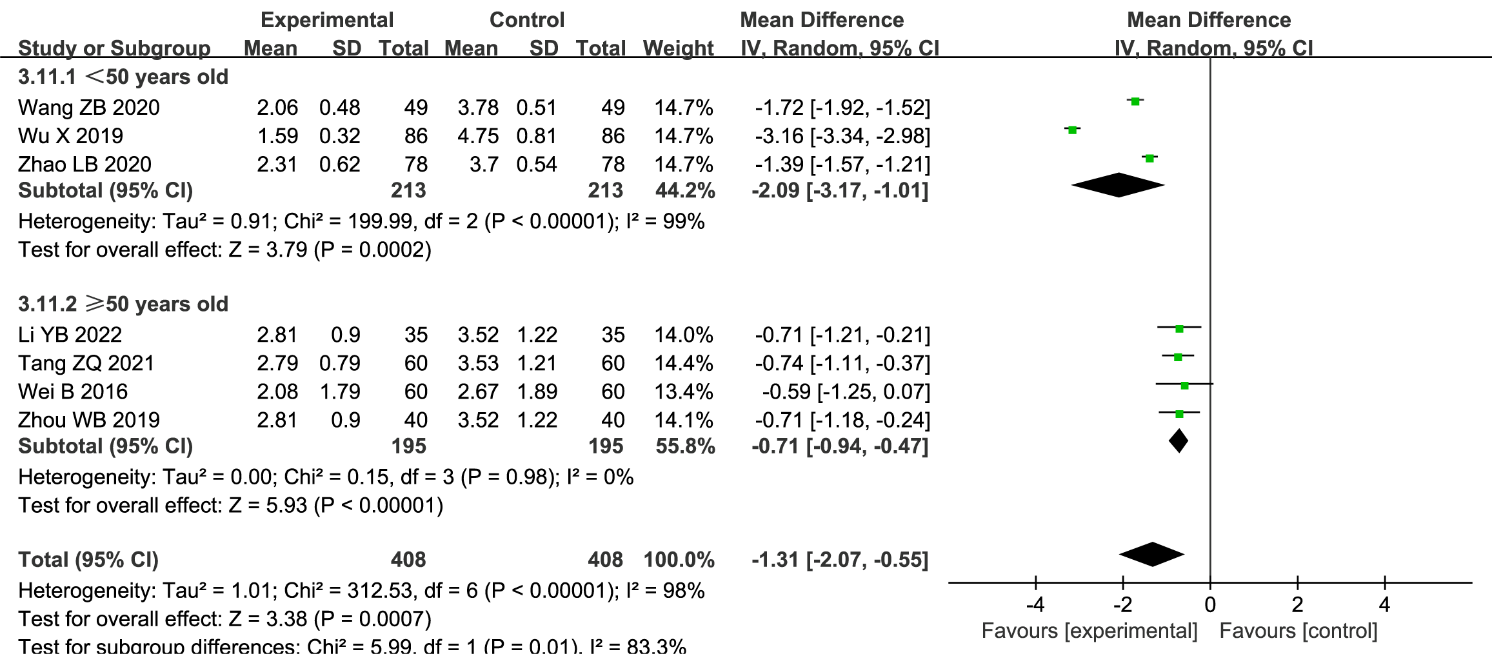


**Supplementary Figure S1.** Subgroup analysis of the Resolution time of fever. (A) Based on the study year; (B) Based on the mean age


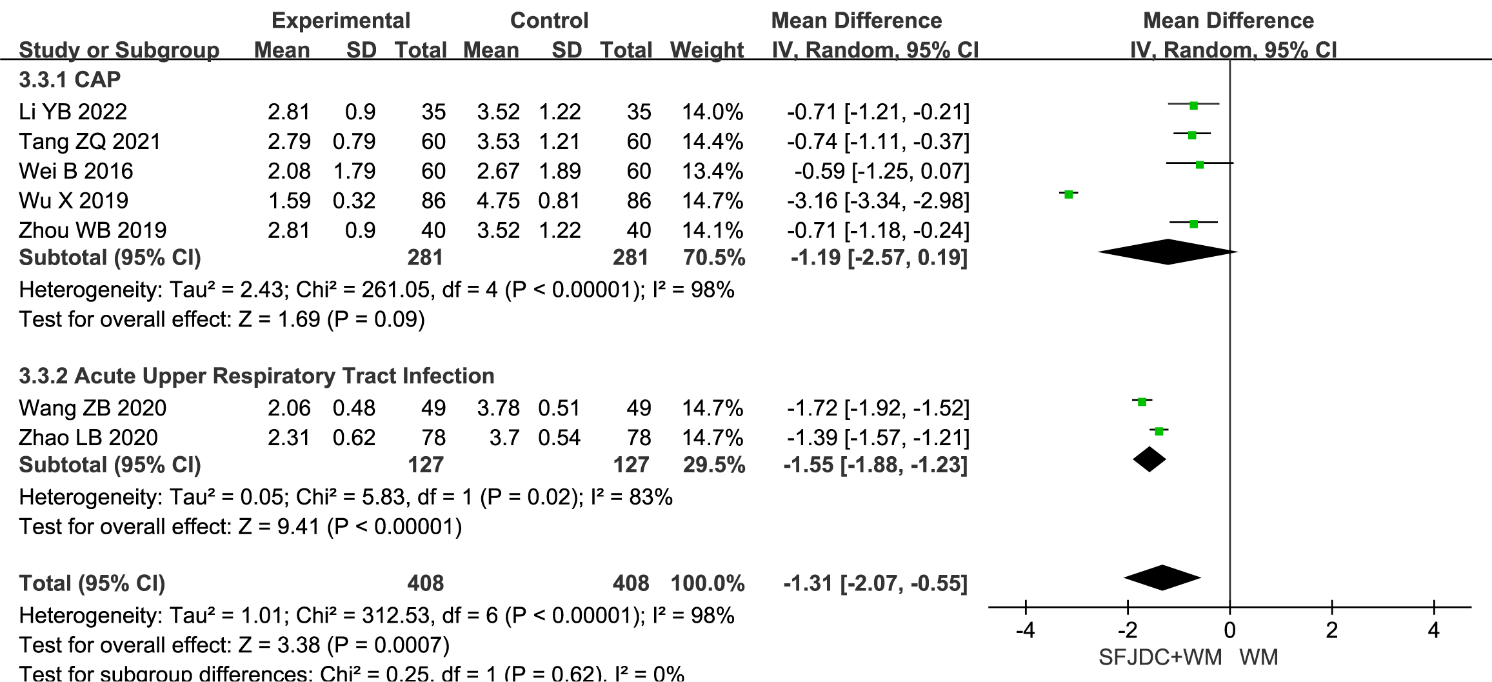


**Supplementary Figure S2.** Forest plot of Resolution time of fever before removal of Wu Xia's study

(A)
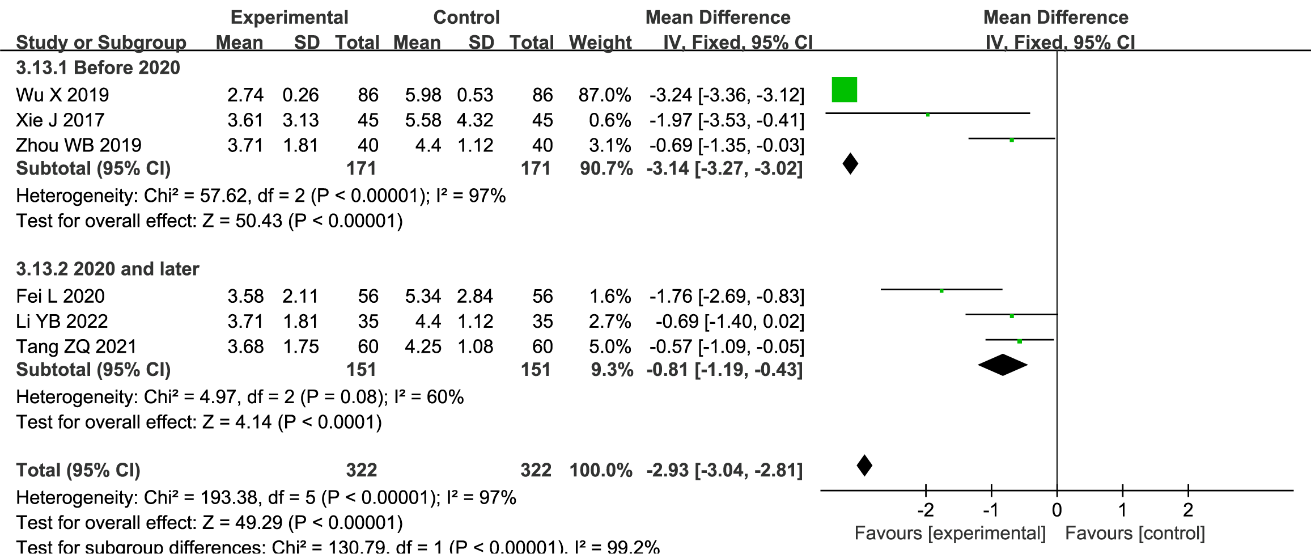


(B)
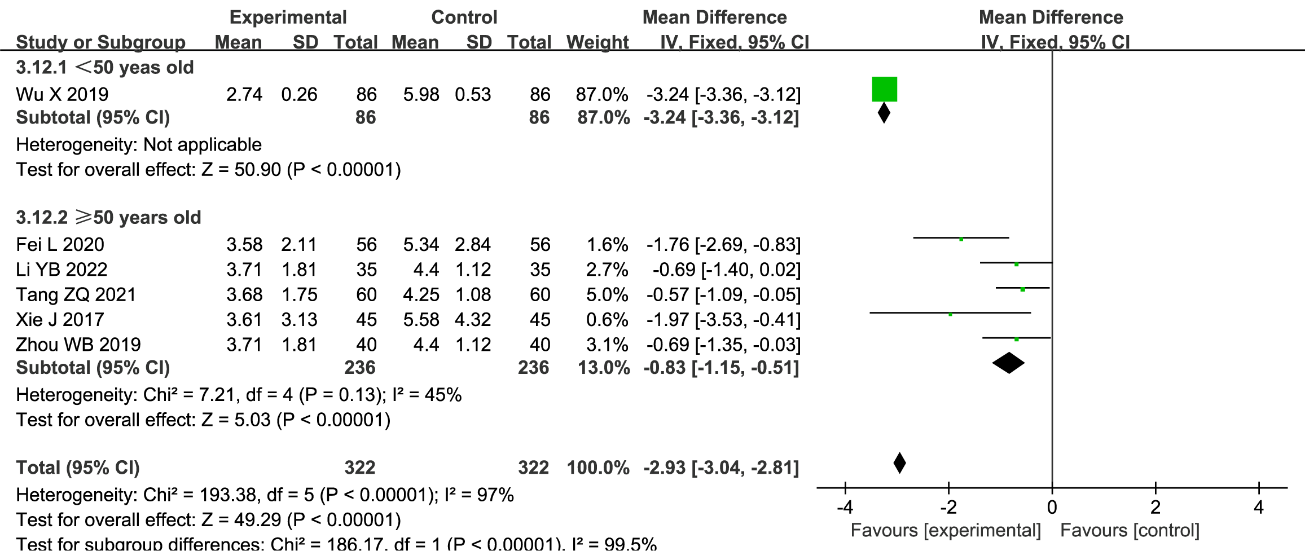


(C)
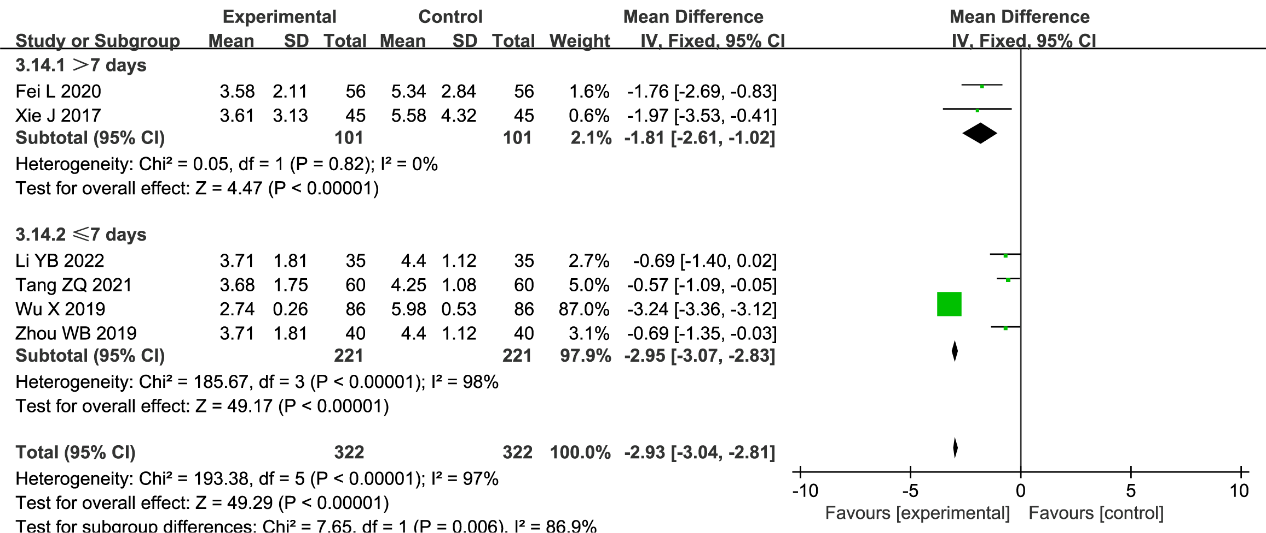


**Supplementary Figure S3.** Subgroup analysis of the Resolution time of pulmonary rales. (A) Based on the study year, (B) Based on the mean age, (C) Based on the course of treatment


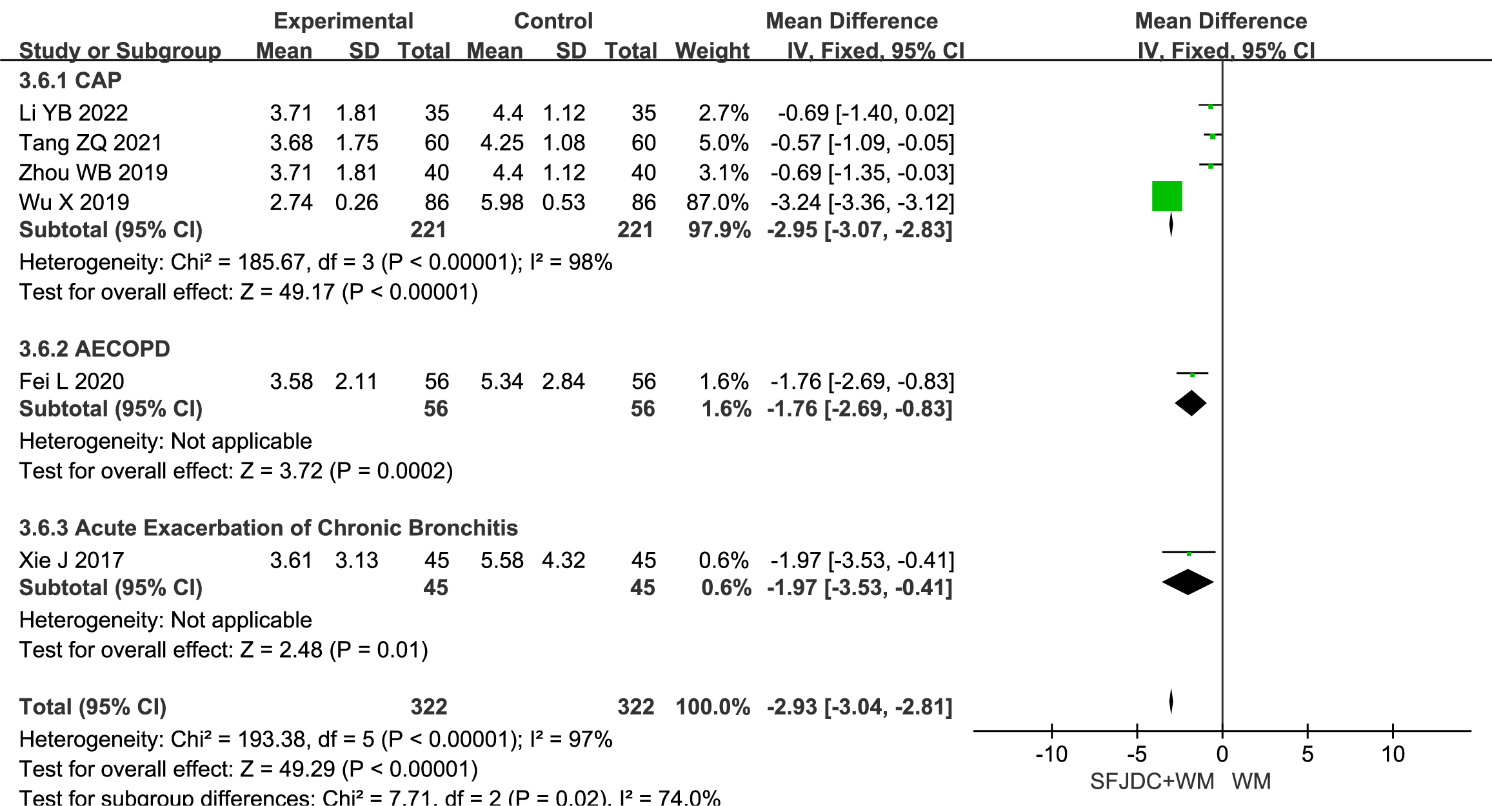


**Supplementary Figure S4.** Forest plot of Resolution time of pulmonary rales before removal of Wu Xia's study

(A)
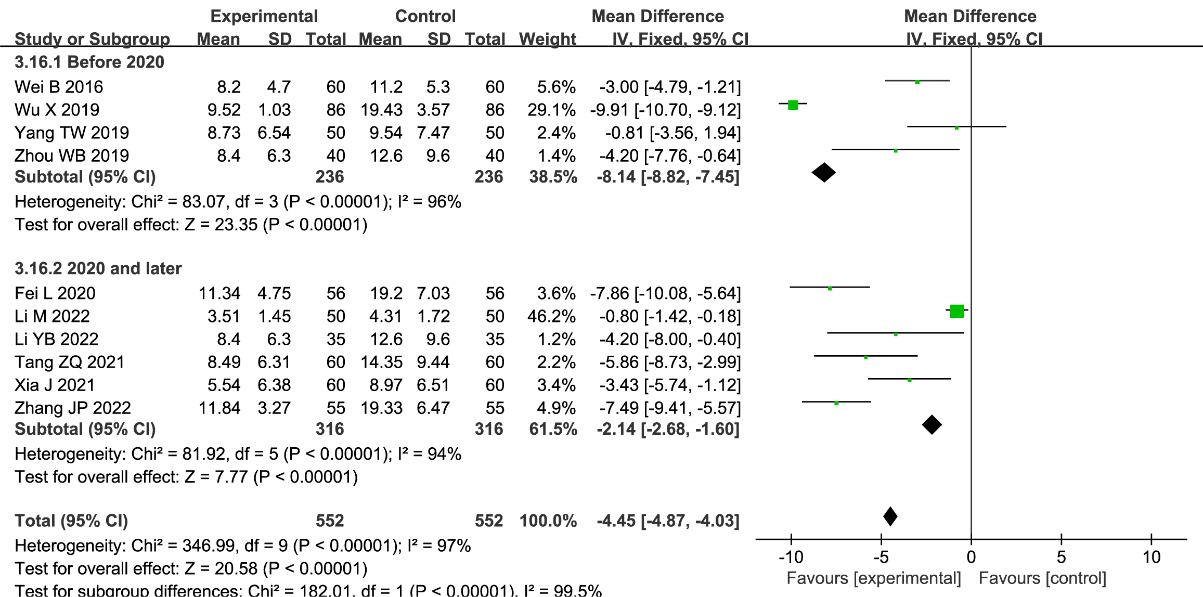


(B)
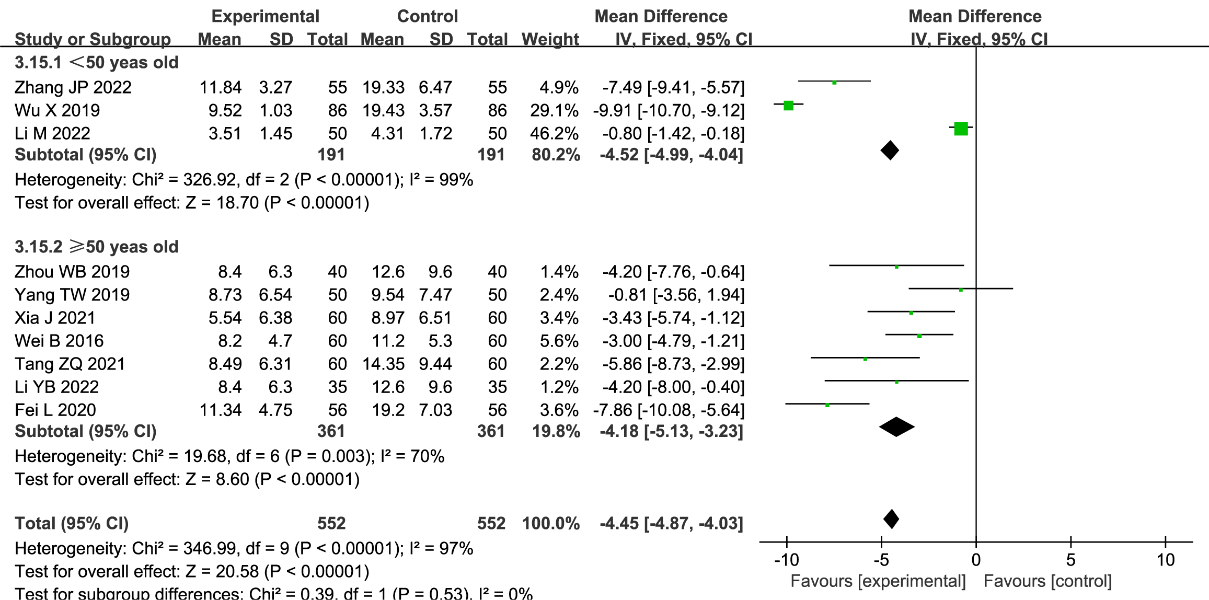


(C)
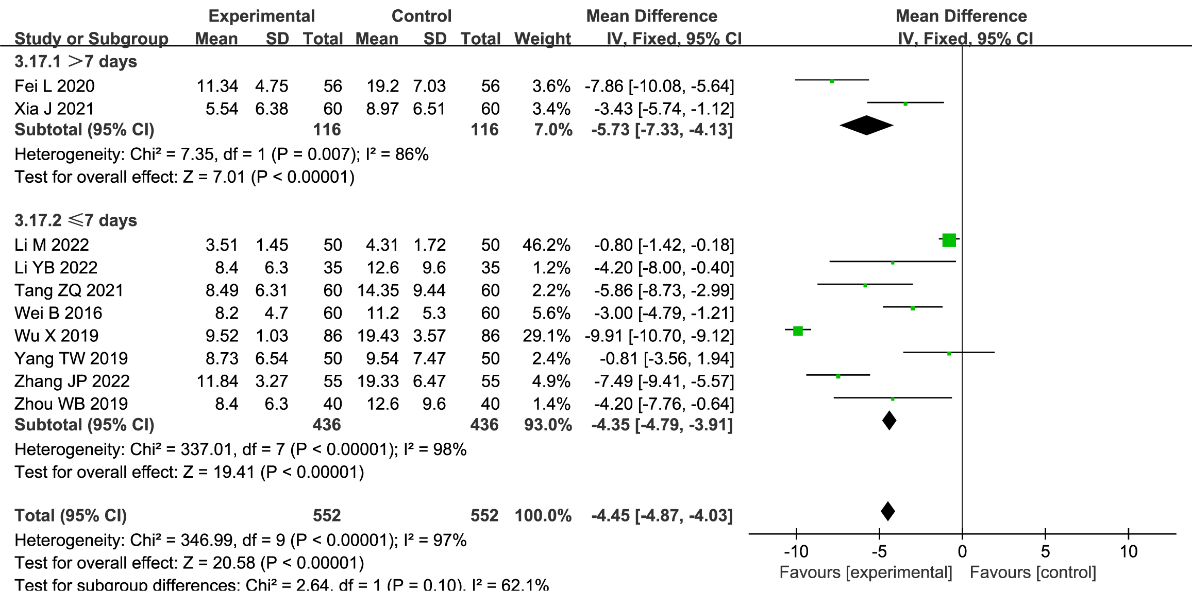


**Supplementary Figure S5.** Subgroup analysis of CRP. (A) Based on the study year, (B) Based on the mean age, (C) Based on the course of treatment


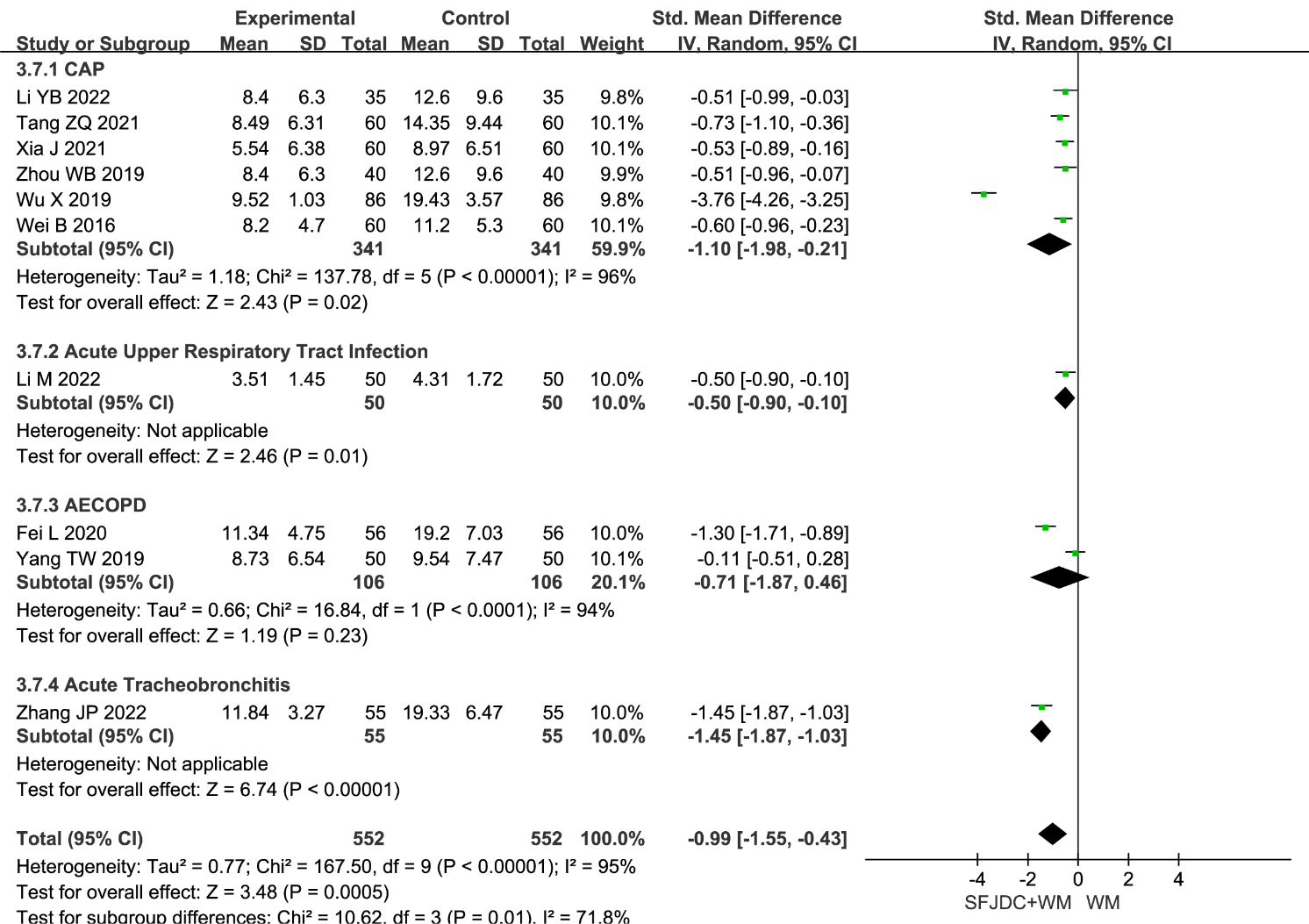


**Supplementary Figure S6.** Forest plot of CRP between experimental group and control group.

**
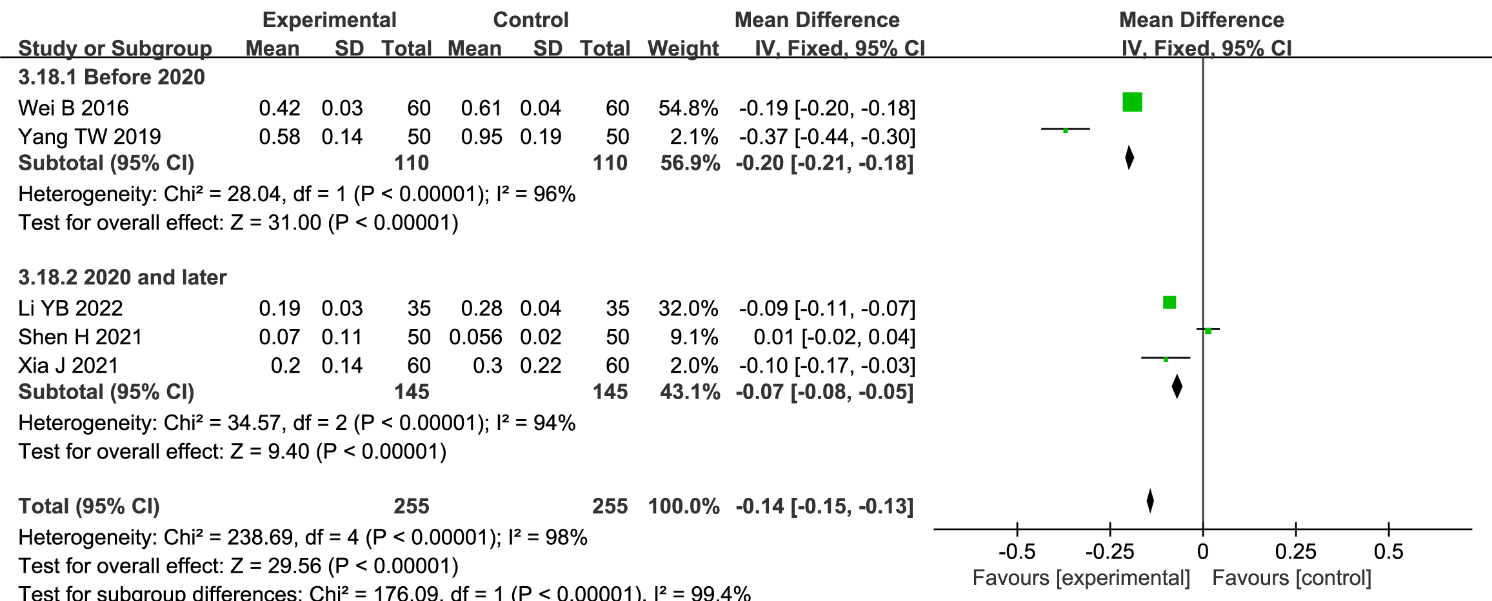
**

**Supplementary Figure S7.** Subgroup analysis of PCT Based on the study year.

**
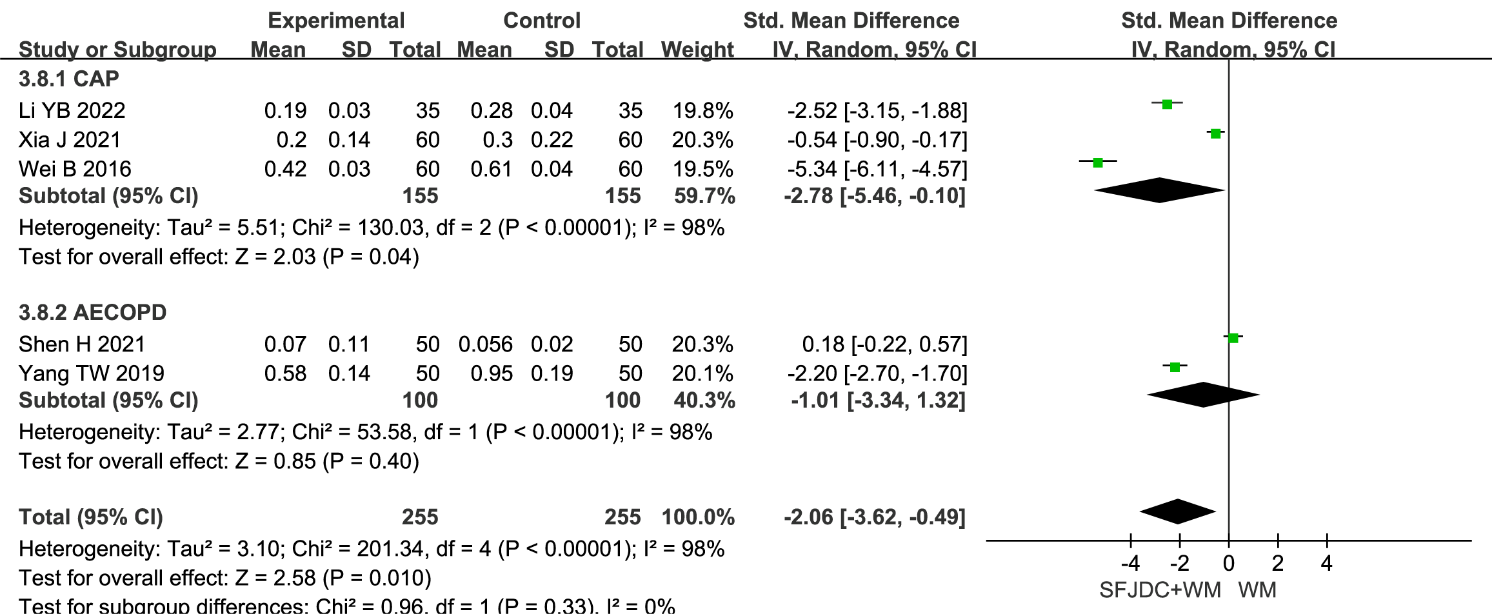
**

**Supplementary Figure S8** Forest plot of PCT between experimental group and control group.

**
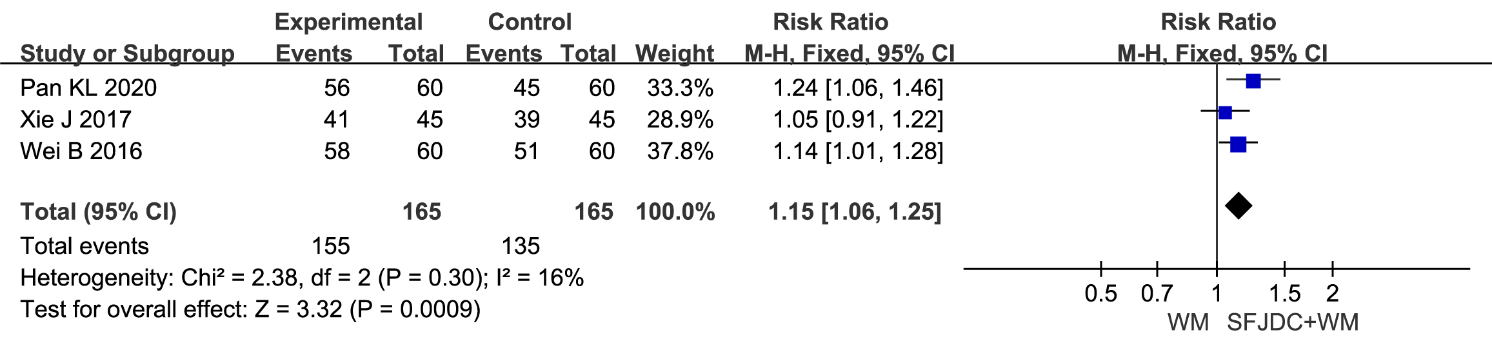
**

**Supplementary Figure S9** Forest plot of Imaging absorption rate between experimental group and control group.

**
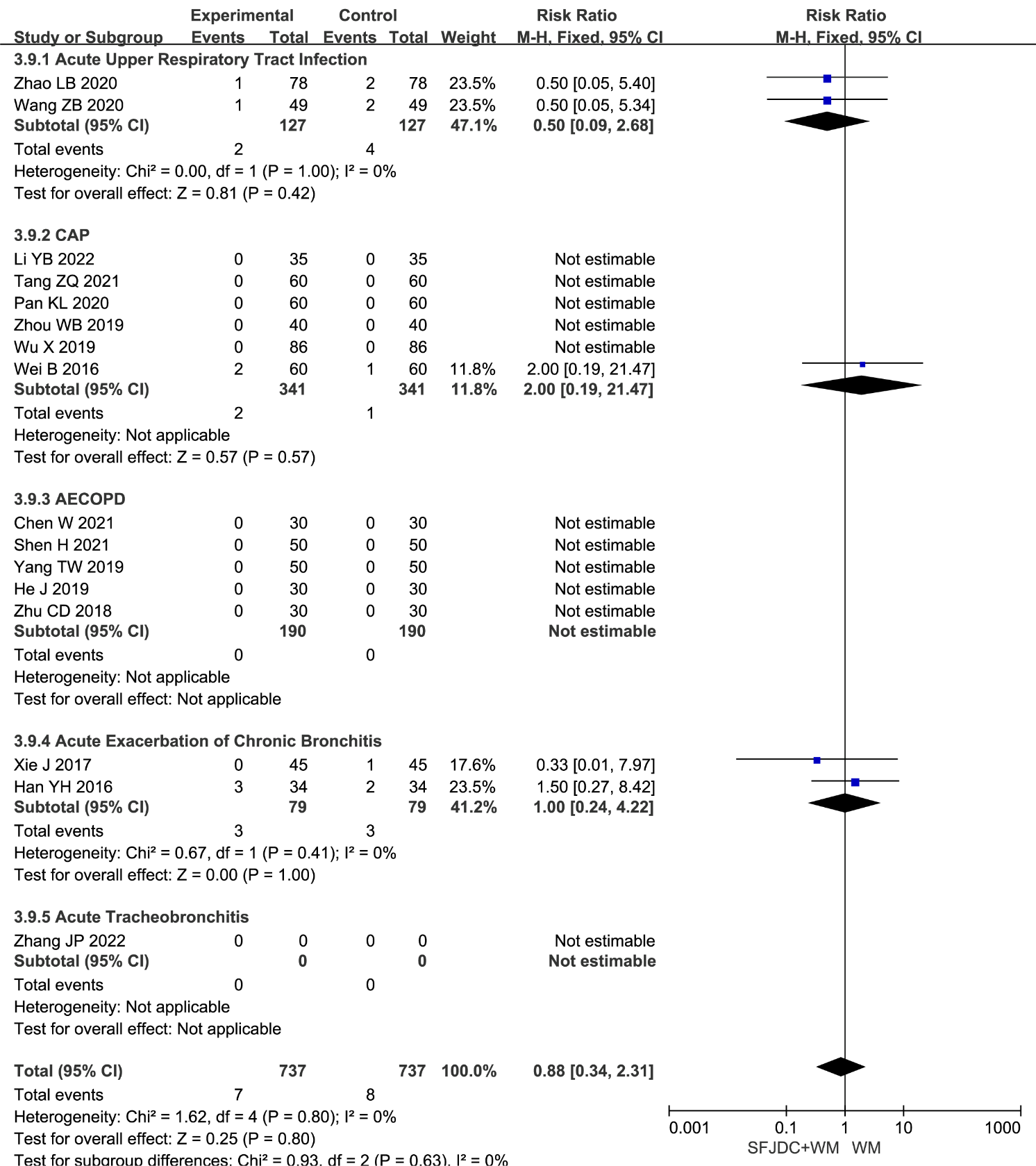
**

**Supplementary Figure S10** Forest plot of Adverse Events between experimental group and control group.

(A)
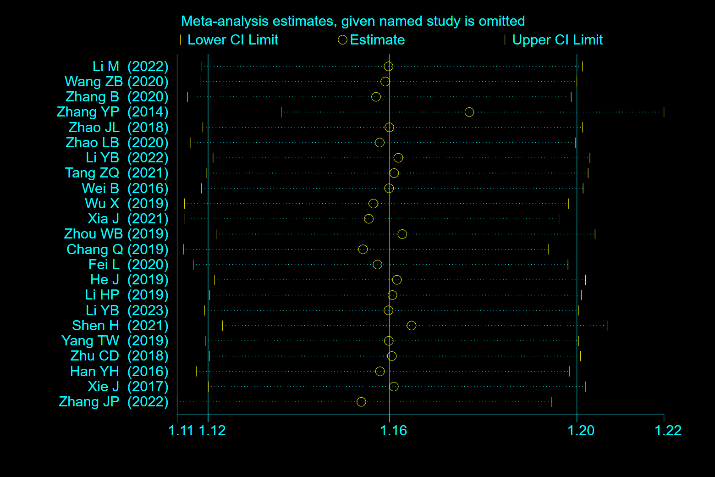
(B)
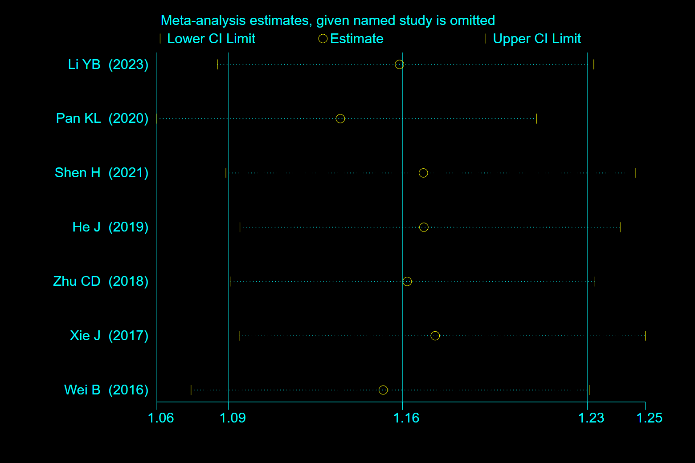


(C)
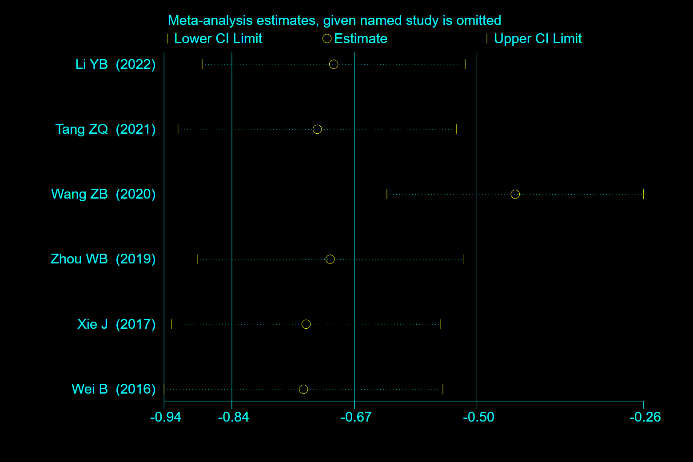
(D)
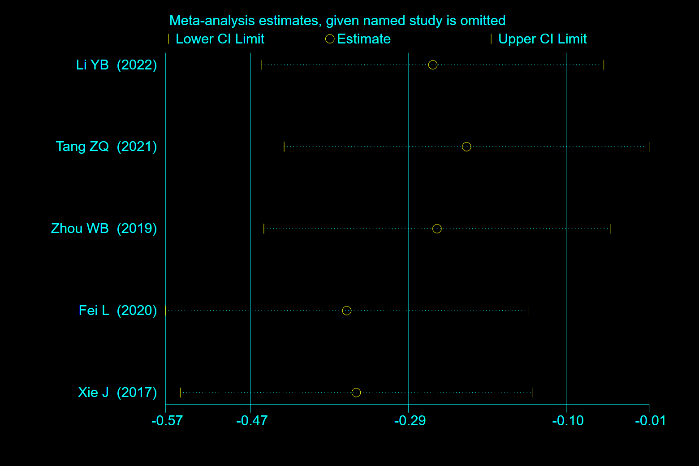


(E)
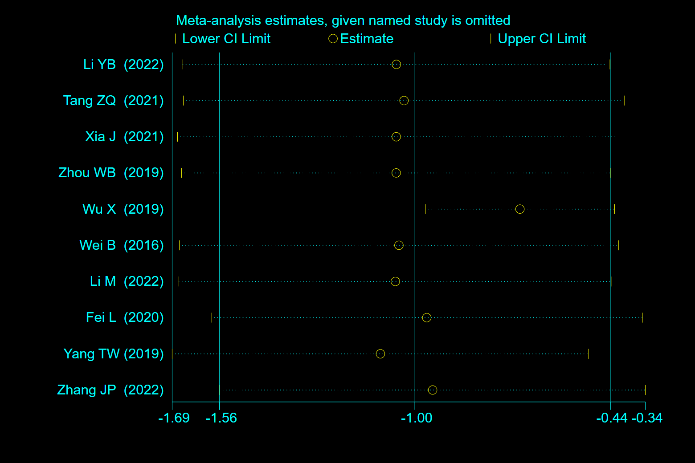
(F)
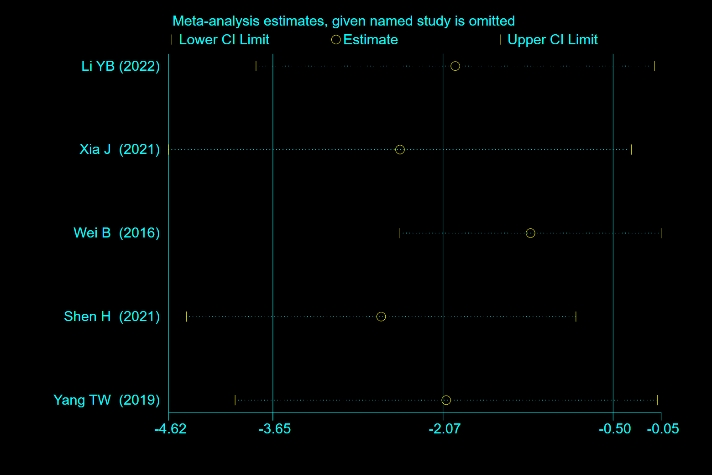


(G)
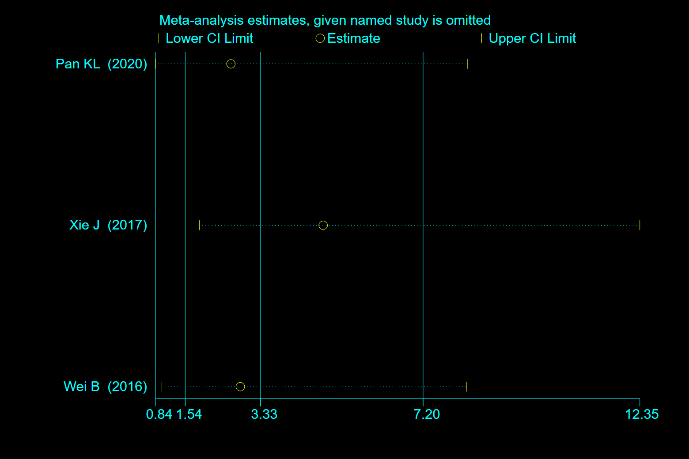
(H)
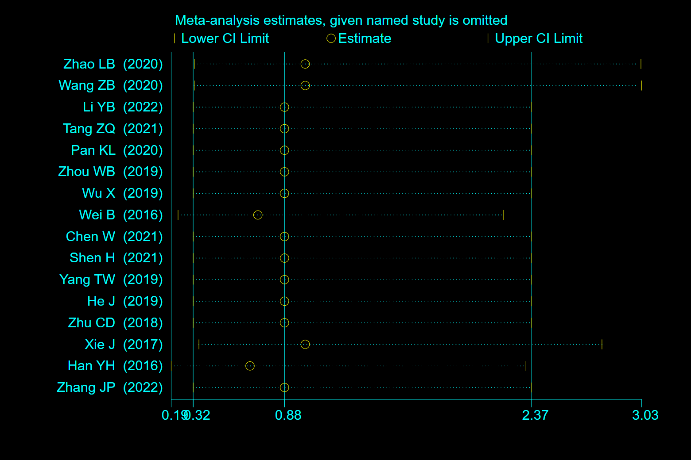


**Supplementary Figure S11.** Sensitivity analysis. (A) Clinical effective rate. (B) Effective rate of TCM syndromes. (C) Resolution time of cough (D) Resolution time of phlegm (E) CRP (F) PCT (G) Imaging absorption rate (H) Adverse Events

(A)**
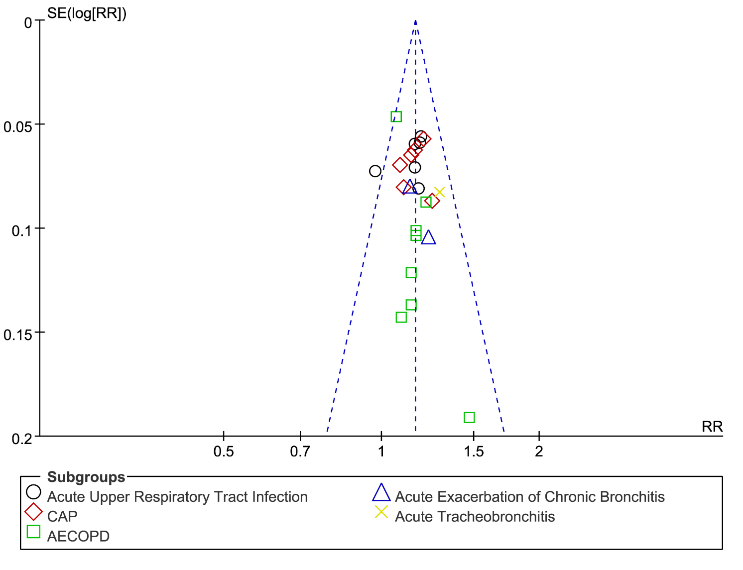
**(B)
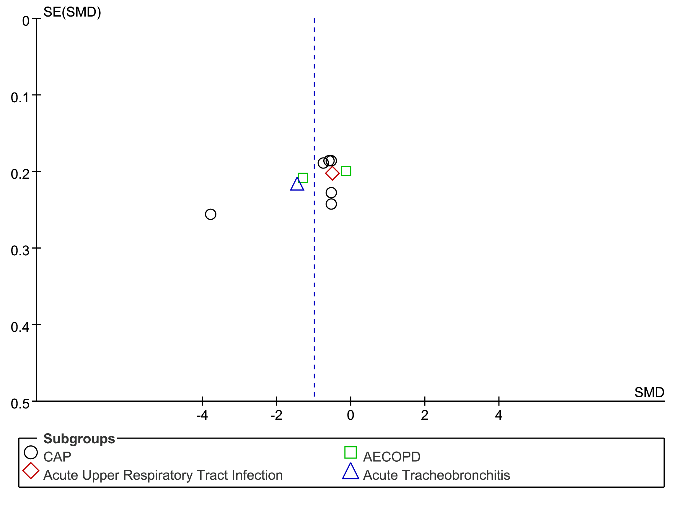


(C)**
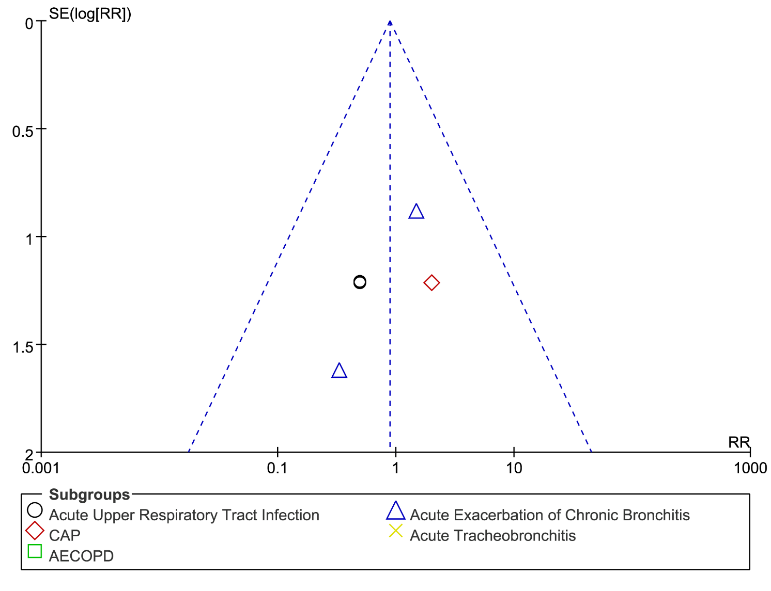
**

**Supplementary Figure S12** Funnel plots of publication bias. (A) Clinical effective rate. (B) CRP (C) Adverse Events

(A)
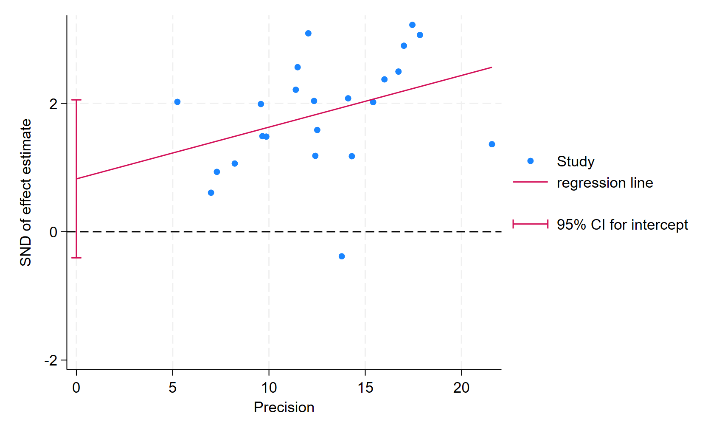
(B)
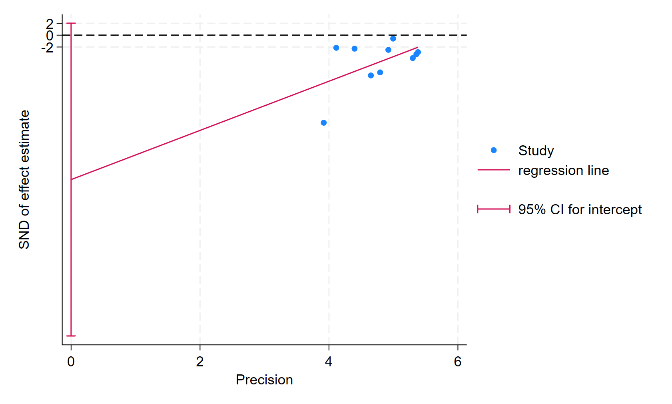


(C)
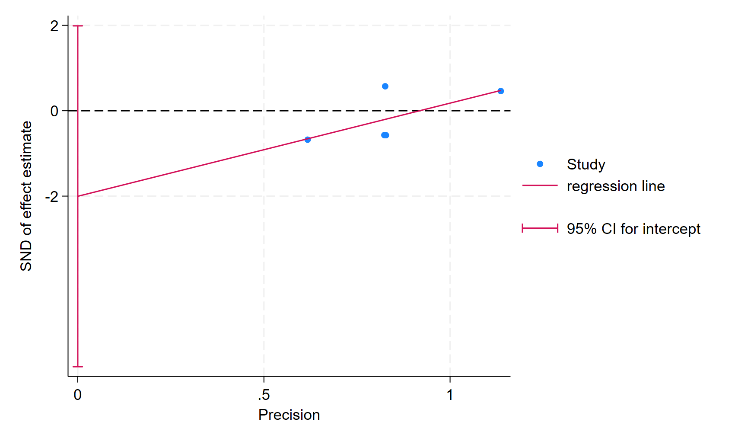


**Supplementary Figure S13** Egger’s plot. (A) Clinical effective rate. (B) CRP (C) Adverse Events
